# Na-Ion Interstitial Site Filling and Structural Evolution in a Tunnel-Structured Vanadium Oxide Insertion Host

**DOI:** 10.1021/acsmaterialsau.6c00206

**Published:** 2026-09-30

**Authors:** James Pérez-Vázquez, Sarbajeet Chakraborty, Yu-Hsiang Chiang, Aysan A. Yilmaz, Victor A. Gomez, Yuting Luo, Leonardo Gobbato, David A. Santos, Cade Alaniz, Denis Sheptyakov, Grigory Smolentsev, Olga Safonova, Maarten Nachtegaal, Mohammed Al-Hashimi, Sarbajit Banerjee, Diana Quintero-Castro, Dominika Baster

**Affiliations:** † Laboratory for Inorganic Chemistry, Department of Chemistry and Applied Biosciences, 27219 ETH Zürich, Vladimir-Prelog-Weg 2, Zürich 8093, Switzerland; ‡ PSI Center for Energy and Environmental Sciences, 28498 Paul Scherrer Institute, PSI, Forschungsstrasse 111, Villigen 5232, Switzerland; § Department of Materials Science and Engineering, 1466 Johns Hopkins University, Baltimore, Maryland 21218, United States; ∥ Department of Chemistry, 14736 Texas A&M University, College Station, Texas 77840, United States; ⊥ Laboratory for Neutron Scattering and Imaging, PSI Center for Neutron and Muon Sciences, Paul Scherrer Institute, PSI, Villigen 5232, Switzerland; # PSI Center for Photon Science, Paul Scherrer Institute, PSI, Forschungsstrasse 111, Villigen 5232, Switzerland; ∇ College of Science and Engineering, 370593 Hamad Bin Khalifa University, P.O.Box: 34110, Doha, Qatar

**Keywords:** Na-ion batteries, electrode material, vanadium oxide, insertion host, single crystal.

## Abstract

The tunnel-structured metastable polymorph ζ-V_2_O_5_ has emerged as a promising insertion host for alkali and multivalent ions, yet the mechanisms governing Na-ion storage remain poorly understood. Here, we combine single-crystal and powder, *operando* X-ray diffraction, neutron scattering, and *ex situ* X-ray absorption spectroscopy techniques with electrochemical measurements to elucidate Na-ion insertion and structural evolution in ζ-V_2_O_5_. Topochemical sodium insertion is limited to approximately 0.27 Na per formula unit, with Na ions initially occupying split seven-coordinate β sites while preserving the tunnel framework. Electrochemical sodiation accesses higher Na-ion stoichiometries and reveals a sequential filling mechanism involving β, β′, and C sites concomitant with Na-ion redistribution and reversible structural distortions. Despite the larger size of Na ions in comparison to Li ions, the tunnel framework remains intact throughout cycling and is recovered upon desodiation. *Operando* diffraction and X-ray absorption spectroscopy reveal that approximately 0.3 Na per formula unit remains trapped after the first discharge process, thereby pillaring and stabilizing the framework. The preinserted pillared phase enables subsequent reversible Na-ion storage. The ability to reversibly insert both Li and Na ions in ζ-V_2_O_5_ provides the rare opportunity to contrast ion insertion and migration mechanisms. Comparison of lithiation and sodiation mechanisms in ζ-V_2_O_5_ demonstrates distinct differences in the thermodynamics and kinetics of ion insertion despite similar insertion sites and ion mobility. These findings provide mechanistic understanding of sodium storage in metastable ζ-V_2_O_5_ and establish design principles for robust tunnel-type cathodes for Na-ion and dual-ion batteries.

## Graphical abstract

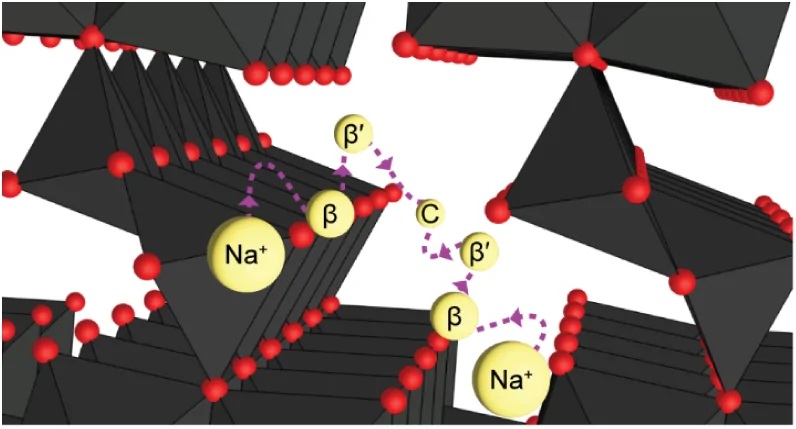

## 1 Introduction

Electrochemical energy storage technologies have been largely dominated by Li-ion batteries (LIBs).^1^ The rapid growth of electromobility and the increasing dependence of power grids on intermittent renewable energy sources have drastically increased demand for energy storage at scale.^2^ However, concerns regarding the sustainability and emerging criticality of Li resources for large-scale applications, such as renewable energy integration and grid-level storage, have sparked interest in alternative battery chemistries such as rechargeable Na-ion batteries (NIBs) to derisk supply chains underpinning grid electrification.^3–6^ In contrast to lithium, sodium mineral sources are abundant, inexpensive, and geographically dispersed. Sodium has a highly negative redox potential (−2.71 V vs SHE), comparable to that of lithium (−3.04 V vs SHE), which makes it an attractive candidate for battery technologies.^7^ Despite these advantages, the mechanisms governing reversibility, kinetics, and preference for Na-ion insertion over parasitic reactions remain incompletely understood, and the available palette of viable insertion hosts is considerably sparser in comparison to Li ions. The larger ionic radius of the Na-ion (1.02 Å when 6-fold coordinated) as compared to that of the Li ion (0.76 Å when 6-fold coordinated) underpins challenges in the design of effective insertion hosts that can accommodate Na ions while sustaining fast and reversible ion insertion/deinsertion kinetics.^7^ As such, deciphering Na-ion insertion mechanisms in terms of reversibility, interstitial site preferences, sequence of site filling, and magnitude of migration barriers, and deriving design principles for new Na-ion battery insertion hosts are crucial to realizing the full potential of Na-ion batteries.

Vanadium oxides have been extensively studied as electrochemical insertion hosts because of their multielectron redox, variable covalency/ionicity of V—O bonds, and diverse structural configurations that define open frameworks with a multitude of accessible interstitial sites.^8^ The single-layered orthorhombic α-V_2_O_5_, which is the thermodynamically stable phase, has been widely studied as an insertion material for LIBs; however, α-V_2_O_5_ presents a wide range of structural transformations, which are irreversible at Li-ion concentrations beyond 1 Li per formula unit, resulting in electrode degradation traceable to the compounding of compositional, phase, and stress heterogeneities.^9–12^ Moreover, the formation of phase boundaries and the stabilization of self-trapped small polarons impede Li-ion diffusion within the electrode, resulting in suboptimal insertion and migration kinetics.^11,13^ Beyond LIBs, the applicability of conventional α-V_2_O_5_ to sodium-ion batteries is limited because of sluggish Na-ion diffusion and structural constraints, resulting in reversible capacities of only 60–80 mAh g^–1^ at a current density of 80 mA g^–1^.^14^ The major bottleneck arises from spatially constrained transition states between interstitial sites and high activation energy barriers for Na-ion migration through the periodic crystal lattice.^14^ The poor cycling stability eventually results in the formation of amorphous Na_2_V_2_O_5_.^15^ In addition to the low capacity and poor stability, V_2_O_5_ also faces barriers of poor electrical conductivity and low working potential as compared to other cathodes of SIBs.^15^

ζ-V_2_O_5_, a recently discovered tunnel-structured polymorph, has shown great promise as a Li-ion battery cathode because the deleterious sequence of gliding transformations characteristic of α-V_2_O_5_ is alleviated.^16,17^ Increasing concentrations of Li ions are instead accommodated through cation ordering along the quasi-one-dimensional tunnel of ζ-V_2_O_5_. Indeed, micrometersized particles and ζ-V_2_O_5_ nanowires deliver approximate initial discharge capacities of 264 mAh g^–1^ and 257 mAh g^–1^ at a current rate of 0.05 C in a lithium half-cell.^18^ Distinctively, Li-ion interstitial sites and diffusion pathways have been traced using a combination of single-crystal-to-single-crystal transformations and first-principles computational studies.^12,19^ Unlike layered orthorhombic V_2_O_5_, the ζ-V_2_O_5_ tunnel structure shows relatively modest volume alteration upon Li-ion insertion. The three-dimensional atomic connectivity of this insertion host imbues superior structural stability, which is critical for long-term battery cycling performance.^18^ When the concentration of Li ions is *x* < 0.33 in Li_*x*_V_2_O_5_, Li ions occupy half of seven-coordinated β interstitial sites.^20^ With increasing concentration of Li ions until *x ∼* 0.66 mol per V_2_O_5_ unit, more closely spaced and five-coordinated β′-sites are occupied, which are shifted by half a unit cell length from the β-sites to alleviate electrostatic repulsion between Li ions.^16,20–22^

Beyond Li ions, a rich diversity of vanadium bronzes and intercalation compounds have been explored through the incorporation of a wide range of cations into the interstitial sites and galleries of V_2_O_5_ tunnel and layered frameworks.^23–26^ Reversible topochemical and electrochemical insertion of Mg^2+^ into ζ-V_2_O_5_ was evidenced, showing a reversible capacity of 90 mAh g^–1^ retained for 100 cycles.^23^ In addition, previous works reported that larger alkali- and alkaline-earth cations occupy β-sites with stoichiometries *x* ≤ 0.33 in M_*x*_V_2_O_5_ (M = alkali, alkaline-earth metals, or transition metals).^26–29^ While ζ-V_2_O_5_ is a versatile insertion host, direct synthesis and hitherto applied topochemical methods yield relatively low insertion stoichiometries. Indeed, understanding of site-filling sequences, structural distortions, and diffusion kinetics at high ion stoichiometries (beyond *x* > 0.33), and means of accessing such regimes, such as through electrochemical approaches, represent a major gap.

Although tunnel-structured β-Na_*x*_V_2_O_5_ has previously been investigated as a cathode material for both NIBs and LIBs,^8,19,30–37^ its crystal structure, electrochemical properties, and ion-storage behavior have primarily been studied in materials obtained through direct chemical syntheses. In contrast, scarce little is known about the structural evolution, site-filling sequences, charge-compensation mechanisms, and mesoscale sodiation heterogeneities of the empty metastable tunnel-structured ζ-V_2_O_5_ polymorph during electrochemical Na-ion insertion. Furthermore, the extent to which electrochemical sodiation accesses intermediate sodium configurations and metastable states that are inaccessible through conventional synthesis routes remains unclear. To the best of our knowledge, no previous study has comprehensively examined how the structure, sodiation heterogeneity, and charge-compensation mechanisms of ζ-V_2_O_5_ evolve upon electrochemical Na-ion insertion/deinsertion. In this work, we contrast structural distortions and electrochemical redox mechanisms occurring in β-Na_*x*_V_2_O_5_ during topochemical and electrochemical sodium insertion/deinsertion. The interstitial sites occupied by Na ions in single crystals and micron-sized particles of β-Na_*x*_V_2_O_5_ were determined using a combination of single crystal X-ray diffraction (SCXRD), neutron powder diffraction (NPD), and *operando* X-ray diffraction (XRD), and the resulting materials were compared with previously reported β-Na_*x*_V_2_O_5_ materials (Table S1). Na-ion mobility within the crystal lattice was examined by quasi-elastic neutron scattering (QENS) and the galvanostatic intermittent titration technique (GITT). The electrochemical performance was evaluated using cyclic voltammetry (CV) and galvanostatic charge/discharge measurements. Sodiation heterogeneities within individual β-Na_*x*_V_2_O_5_ particles were mapped by scanning transmission X-ray microscopy (STXM). Finally, the charge-compensation mechanisms associated with electrochemical sodium insertion/deinsertion were elucidated using *ex situ* X-ray absorption spectroscopy (XAS) acquired at the V K-edge. The results unravel insertion and distortion mechanisms with site filling analogous to Li ions but with substantively different implications for the magnitude of structure distortions and ion mobility. During electrochemical sodiation, an initial complement of Na ions is consumed in irreversible sodiation to create a pillared “pre-inserted” structure;^30^ the pillared pre-inserted structure is subsequently amenable to reversible Na-ion insertion and extraction.

## 2 Experimental Section

### 2.1 Materials Synthesis

#### 2.1.1 Single Crystal Growth

For the synthesis of the β-Na_0.27_V_2_O_5_ single crystals,^30^ a powder sample of β′-Cu_0.64_V_2_O_5_ was prepared by solid-state synthesis by mixing stoichiometric amounts of elemental Cu powder (99% purity, Sigma-Aldrich) and α-V_2_O_5_ (99% purity, Sigma-Aldrich). The mixture was ball-milled in a SPEX CertiPrep ball mill for 30 min using yttrium-stabilized zirconia (YSZ) balls as the milling media, sealed in a fused silica ampule under vacuum, and heated for 36 h at 550 °C in a Thermo Fisher Thermolyne F47900 muffle furnace. After cooling to ambient temperature, the ampules were opened and the black powder contents ground, sealed, and reannealed as previously for another 36 h to ensure compositional homogeneity. Single-crystals of β′-Cu_0.64_V_2_O_5_ were obtained by sealing the prepared powder samples under a high vacuum of <10^–3^ bar in a fused amorphous silica ampule, melting the powders at 825 °C in a programmable furnace (Thermo Scientific Lindberg/Blue M; with UT150 controller), and cooling through the melting point at a rate of 1 °C/h to 550 °C, followed by cooling to ambient temperature under furnace momentum. The resulting lustrous black needle-shaped crystals were stored in air. To obtain ζ-V_2_O_5_ single crystals, the β′-Cu_0.64_V_2_O_5_ crystals were topochemically decuprated using 4 mol equiv of a 0.005 M solution of NO_2_BF_4_ in acetonitrile at room temperature for 3 days.

Topochemical sodiation of as-synthesized ζ-V_2_O_5_ single crystals with retention of crystallinity was accomplished by treating the single crystals (1 mg in mass) with a 2:1 molar excess of a 0.01 M NaI (≥99.5%, Sigma-Aldrich) solution in acetonitrile at room temperature for 3 days.

#### 2.1.2 Micron-Sized Particles

Powders comprising micron-sized particles of β-Na_0.33_V_2_O_5_ were synthesized by a hydrothermal process. Briefly, 6 g of bulk α-V_2_O_5_ (99.6% trace metal basis, Sigma-Aldrich) was dispersed in 77 mL deionized water (Merck Milli-Q EQ 7000, 18.2 MΩ·cm at 25 °C, TOC < 5 ppb); next, 1.4814 g of sodium acetate trihydrate (≥99% ACS reagent, Sigma-Aldrich) were added to the mixture to achieve a Na:V_2_O_5_ molar ratio of 0.33:1. The mixture was then homogenized and transferred into a 100 mL hydrothermal synthesis autoclave reactor with a polytetrafluoroethylene (PTFE)-lined cup. The obtained powder was filtered by vacuum filtration, using a 1.0 μm pore size Nylon membrane filter from Cobetter, thoroughly washed with deionized water, and then dried.

ζ-V_2_O_5_ powder was prepared by a two-step synthesis as previously reported in the literature.^12^ Briefly, β-Ag_0.33_V_2_O_5_ was initially synthesized, followed by leaching of the inserted Ag ions to obtain the ζ-V_2_O_5_ phase. In brief, 6 g of micron-sized particles of α-V_2_O_5_ (99.6% trace metal basis, Sigma-Aldrich) was dispersed in 77 mL deionized water; next, 1.817 g of silver acetate (99%, Thermo Scientific) was added to the mixture aiming for a Ag:V_2_O_5_ molar ratio of 0.33:1. The mixture was then homogenized using an ULTRA-TURRAX high-shear homogenizer (IKA) before being transferred to a 100 mL PTFE-lined hydrothermal autoclave. The vessel was left to react at 220 °C for 72 h in a Binder Model FD 115 convection oven. The obtained powder was filtered by vacuum filtration using 1.0 μm pore size Nylon Membrane filters from Cobetter and thoroughly washed with deionized water. The leaching step consisted of the same above-mentioned autoclave reactors, where the obtained Ag_0.33_V_2_O_5_ powder was reacted with 0.58 M HCl made from 24.17 mL 37% fuming HCl (EMPARTA ACS, Sigma-Aldrich) filled up to 500 mL with deionized water and left to react overnight at 180 °C in a Binder Model FD 115 convection oven. ζ-V_2_O_5_ powders were obtained after another vacuum filtration using a Nylon membrane filter and subsequent washing with a 10 wt % aqueous sodium thiosulfate solution (anhydrous, 98%, Sigma-Aldrich). The recovered powder was manually milled using a mortar and pestle and dried in the Binder Model ED 56 drying oven at 100 °C.

### 2.2 Electron Microscopy (SEM), Energy-Dispersive X-ray Spectroscopy (EDX)

Scanning electron microscopy (SEM) images were acquired using a Zeiss Ultra 55 operated at an accelerating voltage of 12 keV using the SE2 detector, and elemental analysis was performed by energy-dispersive X-ray spectroscopy (EDS) at an accelerating voltage of 12 keV using an Oxford Ultim Extreme silicon drift EDX detector. The samples were prepared on stainless steel stubs by gently pressing the powder onto carbon tape.

### 2.3 Electrochemical Testing

Electrochemical testing of ζ-V_2_O_5_ was performed in sodium-ion half-cells. The coin-type cells were prepared in a glovebox filled with argon (O_2_, H_2_O < 0.5 ppm). The working electrode was prepared by casting the mixture of 70 wt % of active material (ζ-V_2_O_5_), 20 wt % of conductive carbon (Super C65, Imerys), and 10 wt % of binder (poly­(vinylidene fluoride), HSV-900, Arkema) dispersed in *N*-methyl-2-pyrrolidone (Alfa Aesar, anhydrous, 99%) onto an Al foil substrate, followed by drying at 80 °C in a vacuum oven. Sodium metal (15.6 mm diameter, 0.25 mm thickness, Xiamen Aot Electronics Technology Co., Ltd.) was used as the counter electrode and the reference electrode. Celgard 2400–1200 E-A (17 mm diameter, Cytiva Whatman GF/D glass microfiber filter) was employed as a separator. The electrolyte was 1 M NaPF_6_ in a solvent mixture of ethylene carbonate and diethyl carbonate with a volume ratio of 1:1 (battery grade, Sigma-Aldrich).

The cells were galvanostatically discharged and charged in a voltage range of 1.0–3.5 V, 1.50—3.5 V, 1.0–4.0 V, and 1.50—4.0 V using a Bio-Logic electrochemical potentiostat. Cyclic voltammetry (CV) measurements were carried out by a Bio-Logic electrochemical potentiostat at a scan rate of 0.1 mV s^–1^ in the voltage range of 1.50—3.50 V. Galvanostatic intermittent titration technique measurements were performed using a LANHE (CT2001a) battery testing system. The cells were discharged at a 0.05 C rate for 10 min with a 20 min relaxation time between each flux in the voltage range between 1.5—3.5 V. The corresponding *D*_*N* *a*_+ values were calculated and inferred using the following equation:

1 $$D_{Na^{+}}=\frac{4}{\pi \tau }{[\frac{m_{B}V_{m}}{M_{B}S}]}^{2}{[\frac{\Delta E_{s}}{\Delta E_{\tau }}]}^{2}when\tau \ll \frac{h^{2}}{D_{Na^{+}}}$$

where τ is the specific duration of the current pulse, *m*_B_ is the mass of the active material present in the electrode, *M*_B_ is the molar mass of the active material (189.07 g mol^–1^ for ζ-V_2_O_5_ (β-Ag_0.067_V_2_O_5_)), *V*_m_ is the molar volume of the compound (52.67 cm^3^ mol^1^, calculated from the density obtained by Rietveld refinement using GSAS-II, which is equal to 3.59 g cm^-3^ for ζ-V_2_O_5_ (β-Ag_0.067_V_2_O_5_); Figure S1 and Table S2), and *S* is the surface area of the electrode in contact with the electrolyte, Δ*E*_s_ indicates the change in the steady-state voltage of the cell after the relaxation period, and Δ*E*_τ_ is the measured change in the cell voltage within the duration of the applied current pulse.^37,40–42^

For *ex situ* analysis of cycled electrodes, cells were disassembled in an argon-filled glovebox (O_2_, H_2_O < 0.5 ppm) and rinsed thoroughly with dimethyl carbonate (DMC, anhydrous, ≥99%, Sigma-Aldrich), then dried under dynamic vacuum in the glovebox transfer chamber for 2 h.

### 2.4 X-ray Diffraction

For single crystal X-ray diffraction analysis, suitable needle-shaped crystals were selected, placed onto MiTeGen loop pins coated in paratone oil, mounted, and measured under a flow of nitrogen at 100 K. Single-crystal diffraction data were collected on a Rigaku Synergy-S diffractometer equipped with a Rigaku HyPix-6000HE detector and using Mo K_α_ radiation (K_α_ = 0.71073 Å). Structures were solved using intrinsic phasing. Least-squares refinement for all structures was carried out on *F*.^2^ Structural refinement, the calculation of derived results, and generation of electron-density maps were performed using the SHELXTL package of computer programs, ShelXle and Olex2.^43–45^ A Crystallographic Information File for β-Na_0.28_V_2_O_5_ has been deposited in the Cambridge Structural Database and is available for access with deposition number 2566400. The structural model and occupancies of split sites were refined without imposing any mathematical constraints or tethers.

Powder X-ray diffraction (XRD) on the hydrothermally synthesized β-Na_*x*_V_2_O_5_ powder was performed on the 11-ID-C beamline at the Advanced Photon Source (APS) at Argonne National Laboratory (ANL). Powder X-ray diffraction on prepared electrodes of β-Na_*x*_V_2_O_5_ was performed at the ID-31 beamline of the European Synchrotron Radiation Facility (ESRF). Rietveld refinement of the powder XRD pattern was performed using the GSAS II software package.^46,47^

*Operando* powder X-ray diffraction (XRD) was performed on a Bruker D8 Advance instrument in the Bragg–Brentano configuration with a Mo source (Mo K_α_ λ = 0.7107 Å) using an operating voltage of 50 kV and a current of 45 mA using a custom-designed cell with polyimide windows on either side. The electrochemical cell was prepared in an argon-filled (O_2_, H_2_O < 0.5 ppm) glovebox. Free-standing electrodes of the active material were prepared in a 70:20:10 (w/w/w) ratio of active material, carbon Super C65, and polytetrafluoroethylene with a particle size of >40 μm by mixing the components and grinding in an agate mortar and pestle. A thin homogeneous film was formed after ca. 30 min of mixing. The electrodes were cut as prepared to a diameter of 6 mm. The cell was assembled by placing a current collector of Al foil (2 μm, Goodfellow) below the cathode. A sodium metal (ingot, 99.8%, Chemie Brunschwig AG) was used as the anode, and a GF/C glass fiber (Whatman) was used as the separator. A 1 M solution of NaPF_6_ in 50:50 (v/v) EC/DEC (battery grade, Sigma-Aldrich) was used as the electrolyte. Once assembled, the cells were cycled galvanostatically at a current rate of 0.05 C. *Operando* X-ray diffraction measurements were carried out in transmission mode at an operating voltage of 50 kV and a current of 50 mA. The diffraction patterns were collected continuously with an acquisition rate of 600 s. Diffraction data were analyzed using the GSAS-II software package.^46,47^ Background scans were performed in a cell without a cathode. Pawley and Rietveld refinements were performed on representative 1D diffraction data to evaluate the evolution of lattice parameters.

### 2.5 Neutron Powder Diffraction

Neutron powder diffraction (NPD) measurements were performed with a high-resolution powder diffractometer, HRPT, at the Swiss Spallation Neutron Source, SINQ.^39,40^ The instrument was operated in the medium resolution mode, with an incident neutron wavelength of ∼1.494 Å, and the powder pattern was collected at T ∼ 1.5 K. Approximately 2.0 g of the sample was sealed in a vanadium can with a ∼6 mm outer diameter in a He gas glovebox. An oscillating radial collimator suppressed Bragg peaks from the sample environment completely. Rietveld refinement of the NPD data was performed using the GSAS II software package^48,49^ with its internal tables for neutron scattering lengths and form factors, and a modified β-Li_0.43_V_2_O_5_ crystal structure (CCDC #2008970).

### 2.6 Quasi-Elastic/Inelastic Neutron Scattering

Inelastic neutron scattering was performed using the cold triple-axis spectrometer, TASP, at SINQ on a 4.26 g powder sample. A fixed final energy of 3.25 meV was used for all measurements. Spectra were acquired within the temperature range from 2 to 300 K at a fixed scattering angle of 50°. This position was chosen to avoid contributions from Bragg reflections and phonons to the elastic line and quasielastic signal. Data were acquired by counting 100 s per point, in steps of 0.01 meV. Calibration measurements in a pure vanadium sample, which show purely incoherent elastic scattering, indicate an instrumental resolution of 0.08 meV (FWHM).

### 2.7 *Ex Situ* X-ray Absorption Spectroscopy (XAS)

*Ex situ* XAS measurements were performed at the SuperXAS beamline at the Swiss Light Source (SLS) synchrotron facility in Villigen-PSI, Switzerland, using a 2.1 T bending magnet, ∼10^12^ photons/s at 2.7 GeV and 400 mA.^50^ The ζ-Na_*x*_V_2_O_5_ electrodes for *the ex situ* XAS experiments were prepared following procedures described in preceding sections. V K-edge X-ray absorption spectra were collected in quick-transmission mode at room temperature using ionization chambers filled with a 1.0 bar He/N_2_ gas mixture. Energy calibration was performed using a V foil measured simultaneously with the sample and positioned between the second and third ionization mirrors. The beam was collimated using a Si-coated mirror at 2.9 mrad and subsequently monochromatized using a liquid nitrogen-cooled channel-cut Si(111) crystal in QEXAFS mode. The beam was focused on the sample to a spot size of 1 mm × 0.2 mm (H × V) using a Rh-coated toroidal double-focusing mirror. V_2_O_3_ (99.99%, Sigma-Aldrich), VO_2_ (99%, Sigma-Aldrich), and α-V_2_O_5_ (99.6% trace metal basis, Sigma-Aldrich) were used as reference standards to determine the formal oxidation states of vanadium species. The data were processed and analyzed using ProQEXAFS^51^ and the Demeter^52^ software.

### 2.8 Scanning Transmission X-ray Microscopy (STXM)

STXM data were collected at the spectromicroscopy beamline 10-ID1 of the Canadian Light Source. The beamline is equipped with an elliptically polarized undulator source. Incident soft X-rays in the energy range 508—560 eV were focused onto the sample of interest using a Fresnel zone plate, which includes an order-sorting aperture to eliminate undesired diffraction orders. A plane grating monochromator with 500-line mm^–1^ was used for hyperspectral imaging at the V L-edge and O K edge. A dwell time of 1 ms was used for signal acquisition at each pixel. The image stacks were analyzed using aXis2000.^44^ Region-of-interest (ROI) maps were obtained by singular value decomposition (SVD) of the image stack and by using reference spectral components identified within different regions of the stack, as noted in previous work.^11,12,53–57^ This procedure yields a set of spatially resolved composition maps where intensities represent the pixel-wise signal strength of each of the selected components.

## 3 Results and Discussion

### 3.1 Na-Ion Interstitial Site Filling and Dynamics in ζ-V_2_O_5_

To resolve Na-ion interstitial site occupancy at early stages of sodiation and elucidate the structural evolution associated with sodiation in tunnel-structured vanadium oxides, we employed single-crystal X-ray diffraction (SCXRD) on topochemically sodiated ζ-V_2_O_5_. The three-dimensional reciprocal-space information provided by SCXRD enables reliable high-resolution structure solutions, which provide insight into Na-site occupancies and enable precise quantification of subtle sodiation-induced changes in coordination environments, bond lengths, and framework distortions. Figure 1 shows our approach to topochemical sodiation of ζ-V_2_O_5_.

**Figure 1:**
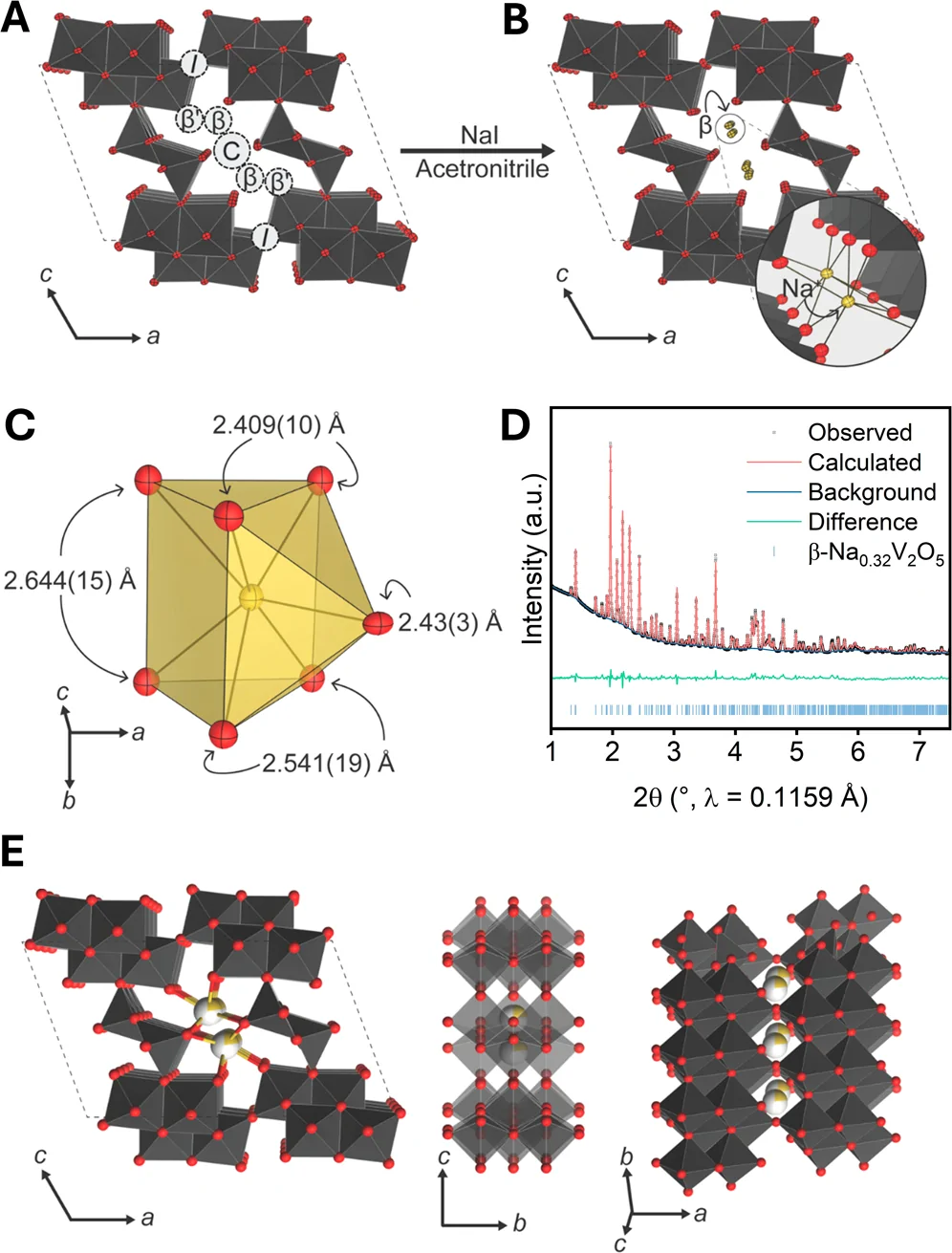
Site filling and structure transformation upon topochemical Na-ion insertion. (a) Crystal structure of the ζ-V_2_O_5_ framework displaying known interstitial sites available for Li-ion occupation. (b) Refined crystal structure of topochemically sodiated β-Na_0.27_V_2_O_5_ determined by single crystal X-ray diffraction (SCXRD) indicating filling of seven-coordinated β sites. (c) Local coordination environment of Na inserted within the β sites as determined from SCXRD. (d) Rietveld refinement of synchrotron XRD data (λ = 0.1159 Å) for hydrothermally synthesized β-Na_0.32_V_2_O_5_ collected at room temperature. Observed, calculated, and difference profiles are shown together with the Bragg reflection positions. (e) Crystal structure of β-Na_0.32_V_2_O_5_ resulting from XRD Rietveld refinement viewed along the *b* crystallographic axis. Red, gray, and yellow spheres represent oxygen, vanadium, and sodium atoms, respectively. Gray polyhedra represent [VO*_x_*] coordination units.

As illustrated in Figure 1A, the ζ-V_2_O_5_ polymorph is constructed from distorted VO_6_ octahedra and VO_5_ square pyramids. Edge-shared V(1)­O_6_ and V(2)­O_6_ octahedra form infinite chains extending along the *b*-axis. These chains connect via shared corners along the *a-*axis to form sheets, which are in turn connected along the *c-*axis by polyhedral corner-sharing with zigzag chains of V(2)­O_5_ square pyramids that define the tunnel walls. Each vanadium ion is off-centered in its polyhedron by the formation of a short covalent bond to one of the surrounding oxygen ions (the “vanadyl” oxygen that forms part of the V(3)­O_5_ square pyramid). The vanadyl oxygens define an interstitial site within the tunnels where Na ions are inserted. As shown in Figure 1C, upon topochemical insertion, Na ions preferentially reside in seven-coordinated β-sites within these tunnels, up to a maximum stoichiometry of *x* = 0.33.^8^

Figure 1B shows that topochemical sodiation of ζ-V_2_O_5_ single crystals yields a *C*2*/m* space group symmetry of the Na-ion-inserted phase with partial occupancies that compute to an overall stoichiometry of 0.27 Na ions per V_2_O_5_ formula unit. Na ions preferentially occupy the split crystallographic β-site with a Wyckoff position 4*i* shown in the inset of Figure 1B; Na-ion insertion in this regime (*x* < 0.33) is accompanied by relatively subtle structural distortions (Tables S3–S7); the monoclinic angle is reduced from β = 110.0° to 109.87° and a modest unit cell expansion is observed from ≈518 Å^3^ to ≈525 Å^3^.^12^ Analysis of the local coordination environments provides a clear rationale for this observed positional disorder. Na_1_ resides in a 7-fold coordination environment with an average Na—O bond distance of 2.5163 Å, primarily coordinated with the vanadyl oxygen atoms. In contrast, a Na_2_ site can be identified, shifted slightly by 0.47 Å along the tunnel path, with a coordination sphere adjusted to a frustrated 7-fold-coordinated environment with an average Na—O distance of 2.518 Å, as shown in the inset in Figure 1B and Figure 1C. Notably, this is in line with Li ions occupying β sites, which are seven-coordinated at low depths of discharge.^18^ This subtle site-splitting reflects a delicate energetic compromise that enables optimal ion—lattice interactions with the tunnel framework while minimizing repulsion of inserted cations along the tunnel. Notably, data collected at 100 K revealed the absence of zigzag ordering of Na ions within the chain; long-range superstructure reflections or *b*-axis doubling characteristic of ion- and spin-ordered systems are not observed (Figure S2), which reflects the Na-ion positional disorder within the framework. Such disorder is distinctive, and indeed yields cation site filling patterns that are differentiated from highly cation-ordered thermodynamic β-Na_*x*_V_2_O_5_ compounds accessed through high-temperature melt growth or hydrothermal synthesis. As such, topochemical Na-ion insertion yields a state of local correlation without the global, long-range crystalline periodicity that emerges from precise Na-ion ordering at the appropriate Na-ion stoichiometries.^58–60^ Such disordered site filling motifs represent metastable configurations as compared to fully ion-ordered systems.^61–63^

Given the limited extent of Na-ion insertion that can be achieved through topochemical reactions in the ζ-V_2_O_5_ framework, β-Na_*x*_V_2_O_5_ nanowires were synthesized using a hydrothermal route. Their morphology is shown in Figure S3. The crystal structure of the resulting material was subsequently characterized by room-temperature (RT) powder X-ray diffraction (Figure 1D and Table S8). Rietveld XRD refinement confirms a Na stoichiometry of *x* = 0.32, with one Na atom occupying the β site. The resulting crystal structure is shown in Figure 1E, and the corresponding refinement parameters are listed in Table S8.

Because X-rays have limited sensitivity to light elements such as oxygen and sodium, while Na ions are expected to exhibit substantial mobility at room temperature, conventional Rietveld refinement of XRD data is insufficient to accurately determine Na occupancies and atomic positions. High mobility of Na ions at room temperature might lead to a distribution of Na density over multiple partially occupied sites and diffusion pathways. To overcome these limitations, neutron powder diffraction (NPD) data were collected at ∼1.5 K, where Na-ion mobility is effectively suppressed. Consequently, the low-temperature NPD data provide a more accurate description of the sodium distribution within the β-Na_*x*_V_2_O_5_ structure. Moreover, neutron diffraction is particularly well suited for locating light elements such as oxygen, refining oxygen atom displacement parameters, and characterizing distortions of the VO_6_ octahedra. Rietveld refinement of the NPD data acquired on hydrothermally synthesized β-Na_*x*_V_2_O_5_ (Figure S4 and Table S9) enabled reliable determination of the Na site occupancies, as illustrated by the structural model shown in Figure S5. Rietveld refinements of the room-temperature XRD data and the NPD data collected at ∼1.5 K yielded highly similar structural models, both retaining 7-fold-coordinated Na ions occupying the β sites identified previously by single-crystal X-ray diffraction. Na insertion at this composition (*x* = 0.32) induces additional structural distortion, reflected by a decrease in the monoclinic angle β from 109.87° to 109.54° as obtained from Rietveld XRD refinement (Table S8). In addition, a modest unit-cell expansion is observed, with the cell volume increasing from approximately 525 Å^3^ (β-Na_0.27_V_2_O_5_ from SCXRD) to 528 Å^3^ (β-Na_0.32_V_2_O_5_ from powder XRD).

The crystal structure of the β-Na_*x*_V_2_O_5_ phase observed in the present study is consistent with the monoclinic β-Na_0.33_V_2_O_5_ structure previously reported in the literature for materials prepared by hydrothermal, sol–gel, solid-state, and soft-chemistry methods (Table S1).^30–39^ Despite differences in synthesis routes, morphology, and sodium content (*x* ≈ 0.28–0.35), these studies consistently report occupation of the characteristic β-type tunnel sites within the monoclinic framework and exhibit closely related lattice parameters (Table S1). Previous studies have relied on direct synthesis methods, in which sodium is incorporated during crystal growth, which enables Na ions to relax to equilibrium positions and occupy thermodynamically stable sites. Consequently, metastable sodium configurations and intermediate structural states generally remain inaccessible through conventional direct synthesis routes. In contrast, topochemical Na-ion insertion into single crystals has not previously been demonstrated. Such an approach enables direct mapping of Na-ion migration pathways and selective occupation of sites located along these pathways.^8,19^ As such, the β-Na_0.32_V_2_O_5_ phase reported here is distinctively stabilized through topochemical sodium insertion into single crystals of ζ-V_2_O_5_ (in subsequent sections, we note sodiated phases derived from electrochemical sodiation of single crystal nanowires in a Na half-cell). The thermal ellipsoids shown in Figure 1 therefore reflect the distribution of Na ions introduced by topochemical insertion and, based on the observed disorder, delineate Na-ion migration pathways and the sequential occupation of interconnected interstitial sites.^8,18,19^ The observed disorder stands, reflecting Na-ion migration along interstitial sites in topochemically sodiated single crystals, is in stark contrast to single crystals prepared by direct melt growth, which show spin-ordering and charge density waves resulting from precise placement of Na-ions in interstitial sites.^58–60^ As reported and discussed below, electrochemical sodiation enables further access to additional metastable sodium configurations that are inaccessible through conventional synthesis methods.

To probe Na-ion diffusion and stochastic motion in β-Na_0.32_V_2_O_5_, quasielastic neutron scattering (QENS) measurements were performed. The resulting QENS spectra are shown in Figure 2. A quasielastic signal with line widths of approximately 0.2 meV is observed at all measured temperatures, confirming the presence of dynamic fluctuations across the entire investigated temperature range. Fitting the spectra requires at least one Lorentzian component. However, slower dynamical processes cannot be resolved reliably within the instrumental resolution of 0.08 meV, as determined from the incoherent scattering of a vanadium standard. The progressive increase in quasielastic intensity with temperature indicates that the observed dynamics are thermally activated. Near room temperature, the relaxation rates exhibit Arrhenius behavior, with activation energies of approximately 0.1 eV ascribed to Na-ion migration barriers in this regime.

**Figure 2:**
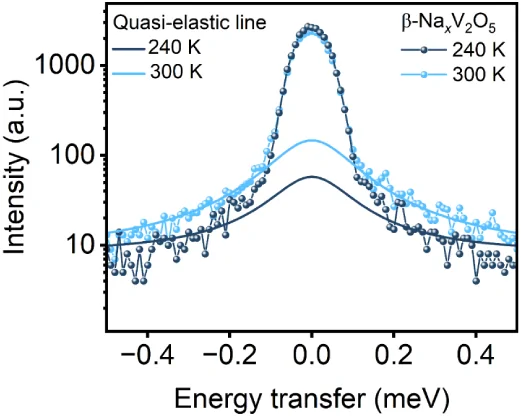
Quasi-elastic neutron scattering and Na-ion migration barriers. Energy scans across the elastic line at a constant wave vector of |Q| = 1.059 Å^–1^ at 240 and 300 K. The intensity corresponds to neutron counts normalized to the monitor counts. The solid lines represent quasielastic components obtained from Lorentzian fits.

### 3.2 Electrochemical Sodium Insertion/Deinsertion into ζ-V_2_O_5_ to Access Higher Na-Ion Stoichiometries

Given that sodium insertion through topochemical and chemical routes is limited to 0.32 Na per V_2_O_5_ formula unit, we next employed electrochemical sodiation of ζ-V_2_O_5_ in sodium half-cells to access higher sodium contents and to explore the viability of ζ-V_2_O_5_ as a positive electrode for Na-ion batteries. Electrochemical insertion enables investigation of the reversibility and Na-site filling sequence at sodium contents beyond *x* = 0.32 and yields a hitherto unexplored class of high-sodium tunnel vanadium oxide bronzes. Cyclic voltammetry and galvanostatic discharge–charge measurements were performed on the as-prepared ζ-V_2_O_5_ powder in sodium half-cells, as shown in Figure 3. During the first discharge process, the galvanostatic profile shows a sloping behavior upon initial sodiation followed by a plateau in the voltage range from 2.0 to 1.5 V, delivering a specific discharge capacity of 170 mAh g^–1^ in the first cycle, which corresponds to sodiation up to Na_1.2_V_2_O_5_. The specific charge capacity in the first cycle is equal to 133 mAh g^–1^, corresponding to 0.9 Na per V_2_O_5_. The lower charge capacity (desodiation process) in comparison with discharge capacity (sodiation process) indicates that ca. 0.3 Na per V_2_O_5_ Na ions are trapped in the crystal structure of ζ-V_2_O_5_ after the first sodiation process, and only part of the inserted Na ions can be reversibly deinserted. Consistent with this picture, electrochemical cycling results demonstrate accompanying significant capacity loss in the first electrochemical cycle from 170 mAh g^–1^ (sodiation process) to 133 mAh g^–1^ (desodiation), indicating the consumption of a fraction (ca. 0.3 Na per V_2_O_5_) of the initial sodium inventory to form the electrode/electrolyte interphase as well as to stabilize a pillared β-Na_*x*_V_2_O_5_ compound. Pillaring Na ions trapped in the crystal structure stabilize a β-Na_0.33_V_2_O_5_ phase in the first cycle, which can then be reversibly cycled (*vide infra*).^30^

**Figure 3:**
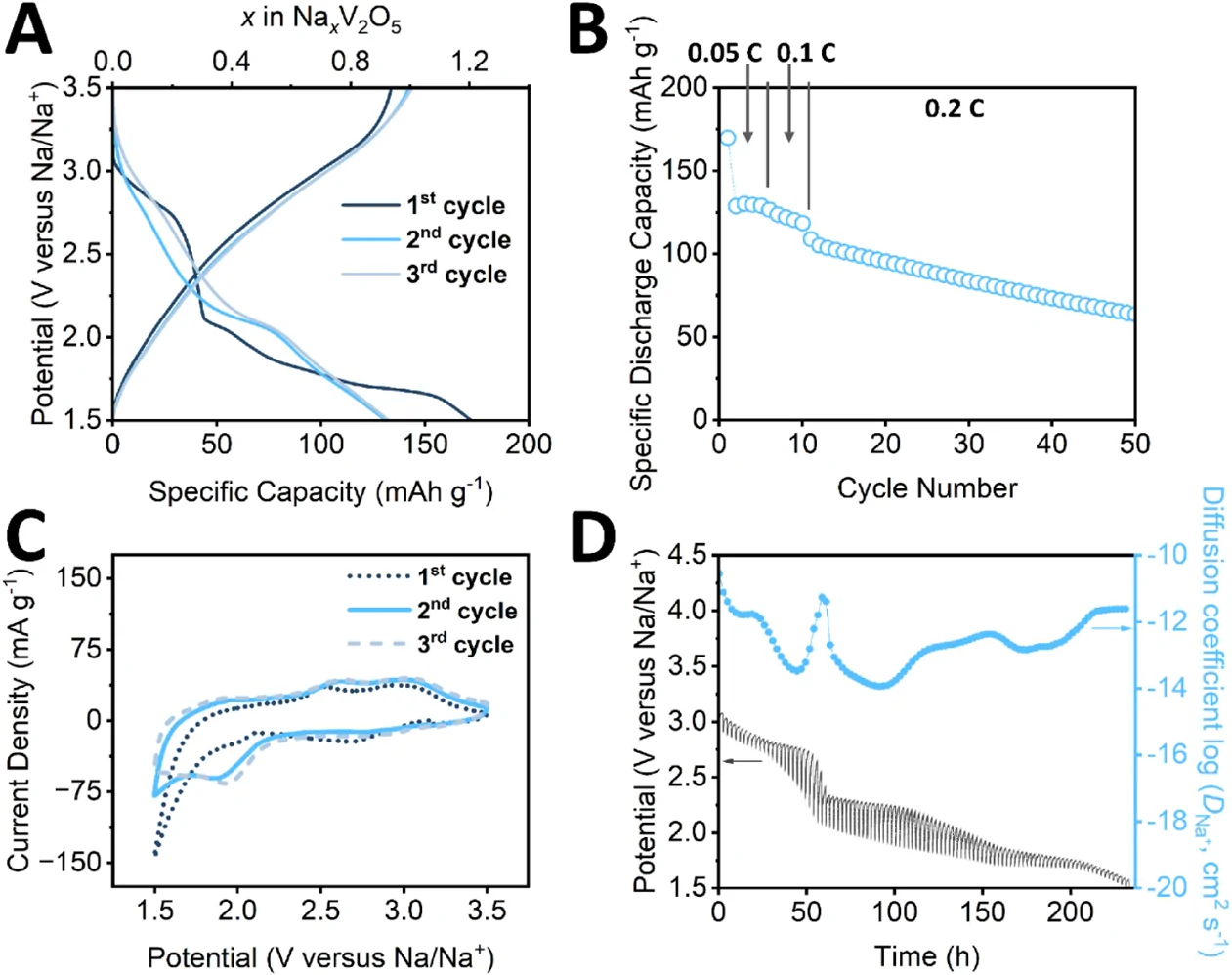
Electrochemical characterization of Na-ion insertion in ζ-V_2_O_5_. (a) Galvanostatic curves of ζ-V_2_O_5_ in the sodium half-cell at a current rate of 0.05 C in the potential range of 1.5—3.5 V. (b) Long-term cycling performance of ζ-V_2_O_5_ at current rates of 0.05, 0.1, and 0.2 C in the voltage range of 1.5—3.5 V. (c) Cyclic voltammograms of a ζ-V_2_O_5_ electrode in the sodium half-cell during three cycles at a 0.1 mV s^–1^ scan rate in the potential range of 1.5—3.5 V vs Na/Na^+^. (d) GITT curves of ζ-V_2_O_5_ plotted alongside inferred sodium diffusion coefficients as a function of the state of charge.

In subsequent cycles, the specific discharge and charge capacities are maintained between 130 mAh g^–1^ and 140 mAh g^–1^. As shown in Table S10, the specific capacities and operating voltage window observed for the β-Na_0.33_V_2_O_5_ obtained through electrochemical sodiation in the Na half-cell are consistent with values previously reported for β-Na_0.33_V_2_O_5_ obtained through conventional direct synthesis methods such as solid-state reactions and hydrothermal synthesis.^30,32,35^ The initial specific-capacity loss is significantly reduced when the hydrothermally synthesized β-Na_0.32_V_2_O_5_ is cycled directly, decreasing from 23.5% for ζ-V_2_O_5_ to 19.9% for β-Na_0.32_V_2_O_5_, because prepillaring consumes additional sodium from the cell inventory (Figure S7). Beyond the first cycle, differences in long-term cycling stability also become apparent. Upon extended cycling, ζ-V_2_O_5_ exhibits capacity fading of approximately 40% between the 10th and 50th cycles (Figure 3B and Figure S6B). In contrast, hydrothermally synthesized β-Na_0.33_V_2_O_5_ shows good cycling stability, retaining 83.3% of its capacity at 50 mA g^–1^ after 100 cycles (Table S10).^35^ The capacity fading of ζ-V_2_O_5_ upon extended cycling can be attributed to the combined *effects of* (i) progressive surface degradation and cathode-electrolyte interfacial reactions during extended cycling, as widely reported for oxide-based cathodes in Na-ion batteries, evidenced also in STXM measurements (*vide infra*),^64–68^ and (ii) pronounced structural distortion arising under deep-sodiation conditions (*x*_Na_ ≈ 1.0), the high-capacity regime investigated in the present work, which gives rise to chemo-mechanical degradation.^18^ For instance, Qiao et al. demonstrated the formation of electrochemically inactive reduced species at cathode surfaces upon cycling,^66^ whereas Mu et al. highlighted the critical role of cathode-electrolyte interfacial degradation in capacity loss.^67^ More recently, Baster et al. directly correlated surface chemical degradation observed by X-ray spectroscopy with electrochemical fading in layered oxide cathodes for Na-ion batteries.^68^

The cyclic voltammetry plot of the first reduction process shows a broad peak at around 2.0 V, which is consistent with the voltage of the plateau appearing in the discharge–charge profile, along with a sharp reduction peak at 1.5 V, suggesting the occurrence of a set of transformations upon Na-ion insertion. Compared to the first cycle, in the subsequent cycles the reduction peak at 2.0 V broadens and increases in intensity, whereas the peak at 1.5 V decreases in intensity. This is accompanied by the loss of the voltage plateau below 2.0 V in subsequent cycles, and a transformation to a sloping character of the voltage profile in the voltage range of 1.5—2.0 V. As such, these results corroborate the insertion of Na ions during the first cycle, consistent with the concomitant capacity loss.

To assess Na-ion diffusion kinetics, galvanostatic intermittent titration technique (GITT) measurements were performed. The Na-ion diffusion coefficient, determined from GITT measurements, is plotted as a function of Na-ion concentration in Figure 3D. During the initial sodiation process, sodium diffusion coefficient values slightly decreased from 10^–11^ to 10^–13^ cm^2^ s^–1^, which is likely ascribed to electrostatic repulsion between inserted Na ions. Notably, between Na-ion concentrations of *x* = 0.3—0.5, there is an increase in Na-ion diffusivity, likely derived from accessing a new complement of interstitial sites within the tunnel framework, as will be discussed in subsequent sections. With still greater Na-ion insertion, the diffusivity is further suppressed to 10^–14^—10^–13^ cm^2^/s because of electrostatic repulsions between ever more closely packed Na ions arrayed in interstitial sites across the tunnel.

To monitor the structural evolution of ζ-V_2_O_5_ during electrochemical sodium insertion and deinsertion, *operando* XRD measurements were performed. Figure 4A (left) shows the first-cycle galvanostatic discharge–charge profiles of a ζ-V_2_O_5_ electrode in a sodium half-cell. Approximately one Na-ion per formula unit is inserted upon discharge to 1.5 V at a current rate of 0.05 C, whereas approximately 0.3 Na ions per formula unit remains irreversibly trapped within the crystal structure after charging to 3.5 V. This behavior is likely a result of irreversible sodiation, as suggested by the low diffusion coefficient values (Figure 3D), and the stabilization of the β-Na_0.33_V_2_O_5_ compound, which resides on the convex hull. The preinserted and pillared β-Na_*x*_V_2_O_5_ compound (*x ∼* 0.33) with an expanded tunnel framework shows outstanding reversible Li-ion insertion/deinsertion,^30^ and, in this case, mediates reversible sodiation/desodiation to higher Na-ion stoichiometry regimes.

**Figure 4:**
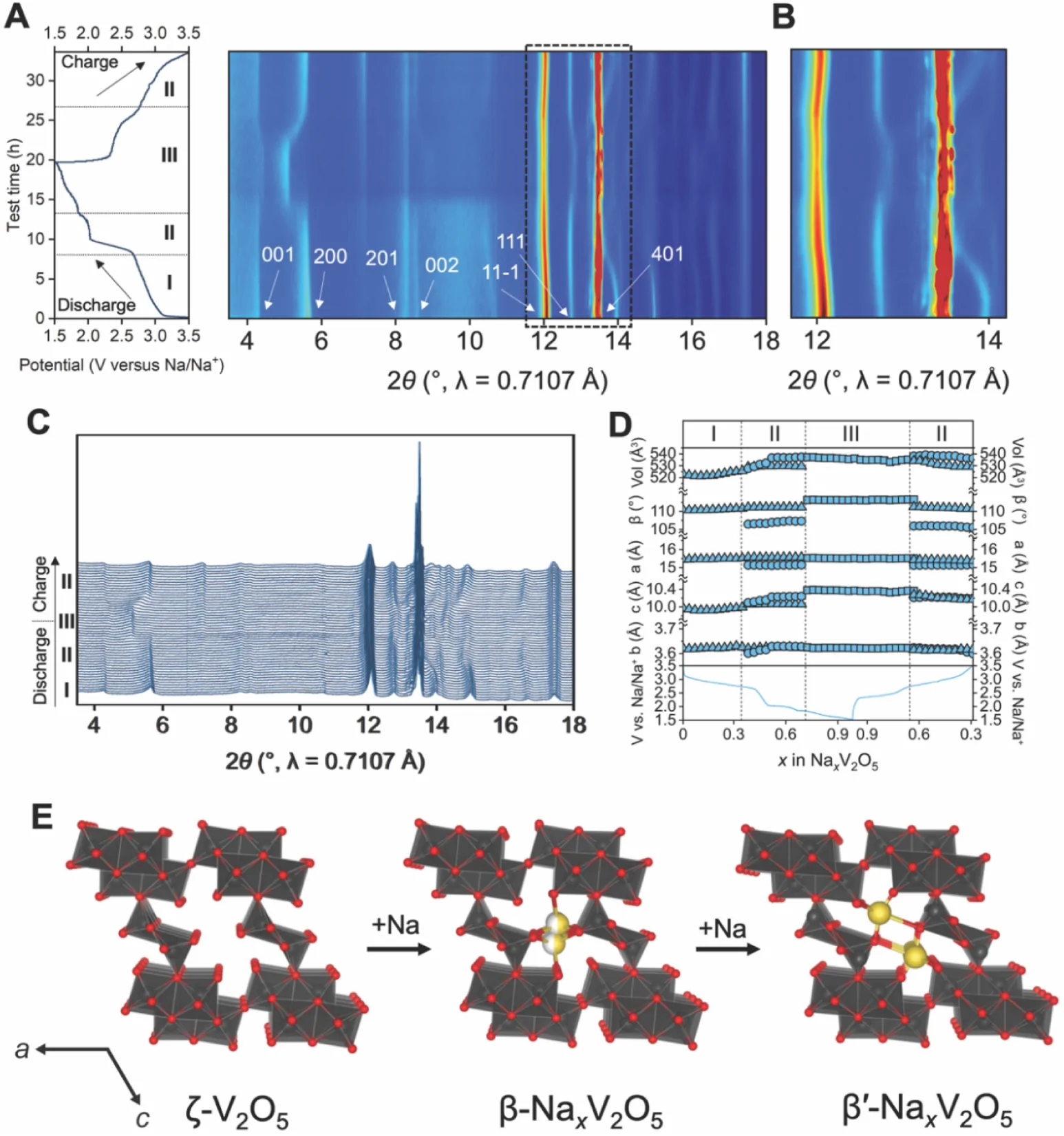
Operando X-ray diffraction study of the structural evolution of ζ-V_2_O_5_ during electrochemical sodium insertion and deinsertion. (a) Galvanostatic discharge–charge profiles (left) acquired during *operando* XRD measurements of ζ-V_2_O_5_ electrodes at a current rate of 0.05 C in the voltage range 1.5—3.5 V vs Na/Na^+^, together with the corresponding contour plot of diffraction intensities (right). (b) Enlarged contour plot showing the evolution of diffraction peak intensities in the 2θ range 11.7—14.2°, as highlighted in panel (a). (c) Waterfall plot of selected diffraction patterns acquired during sodiation and desodiation of ζ-V_2_O_5_, illustrating the redistribution of Na ions among distinct tunnel sites. (d) Evolution of lattice parameters and unit cell volume obtained from Pawley refinement of the XRD patterns shown in panel (c), plotted as a function of *x* in β-Na*_x_* V_2_O_5_ during electrochemical sodiation of ζ-V_2_O_5_. Here, *Vol.* denotes the unit cell volume and β the angle between the *a* and *c* crystallographic axes. (e) Crystal structures of ζ-V_2_O_5_, β-Na*_x_* V_2_O_5_, and β′Na*_x_* V_2_O_5_. Red, gray, and yellow spheres represent oxygen, vanadium, and sodium atoms, respectively. Gray polyhedra represent [VO*_x_*] coordination units.

Based on the contour plot of reflection intensities obtained from the *operando* XRD measurements, three characteristic sodiation/desodiation regimes can be identified, as shown in Figures 4A (*right*) and 4C. These regimes are also consistent with distinguishable slopes and a plateau in the discharge/charge curve. It is worth mentioning that the monoclinic *C*2/*m* space group of the tunnel framework is preserved during the first stage of the discharging process without distortive structural phase transformations.^12^ However, as a result of the large size of Na ions, the second and third regimes induce a stronger structural distortion that reduces the crystal symmetry to the *P*2/*m* space group while still preserving the overall connectivity of the tunnel-structured V_2_O_5_ framework. The observed greater structural distortion is distinctively and substantively amplified as compared to scenarios with Li-ion insertion reported in previous work.^18^ Nevertheless, the highly distorted III regime is phase reversible, which indicates the robustness of this 3D tunnel interconnected framework (Figure 4B).^23^ Figure 4D shows the evolution of the monoclinic β angle and lattice parameters as a function of sodium content in ζ-V_2_O_5_, delineating the distinctive occupancy regimes. During initial discharge to 2.73 V, a slight tunnel expansion is observed in regime I (0 < *x* < 0.33 in β-Na_*x*_V_2_O_5_), ascribed to Na ions occupying seven-coordinated β sites (Wyckoff positions: two of 4i from SCXRD) within the tunnel, which induces a volume change from 523.14 Å^3^ to 525.12 Å^3^ and a β angle change from 110.40° to 110.80°. During discharge to 1.85 V, the Na ions access regime II with 0.33 < *x* < 0.66 in β/β′-Na_*x*_V_2_O_5_, where two Na ions can be refined as occupying both seven-coordinated β and five-coordinated β′ sites (Wyckoff positions: two of 4i from powder XRD) within the tunnel framework of ζ-V_2_O_5_. The greater concentration of Na ions and their positioning across the two distinctive interstitial sites expands the tunnel further to a volume of 537.25 Å^3^ when the β sites are entirely filled, which represents a still modest approximately 2.7% volume expansion compared to empty ζ-V_2_O_5_.

In regime III, corresponding to the end of the discharge process to 1.5 V, 0.66 < *x* < 0.98 in β-Na_*x*_V_2_O_5_, C sites (Wyckoff 2b sites) labeled in Figure 1 start to get occupied, and Na-ion reordering further proceeds within the interstitial sites from β to β′ sites, which minimizes Na—Na electrostatic repulsions and results in a slight contraction of the unit cell to a volume of 536.08 Å^3^. The strong distortion reduces the symmetry of the crystal lattice to the *P*2/*m* space group. Concurrently, severe distortion is observed in Regime III, leading to further changes in the lattice parameters and space group. A plausible structural model is provided from the refinement to the powder XRD pattern in Figure S8. Remarkably, upon charging, the tunnel framework exhibits excellent reversibility, enabling Na-ion reordering from β′/C sites to β/β′ occupancies and restoring the unit-cell parameters of regime II. However, approximately *x ≈* 0.3 Na are not extracted from the crystal structure of β′-Na_*x*_V_2_O_5_ at the end of charging at 3.5 V, which indicates a thermodynamic minimum at this distinctive occupancy, which corresponds to β-site filling.^20^ It is worth mentioning that no layered V_2_O_5_ or related layered phases are observed during the de/sodiation process, which indicates the robustness of the metastable tunnel framework in accommodating Na ions without reversion to the thermodynamically stable α-V_2_O_5_ polymorph.^69^

### 3.3 Charge Compensation Mechanisms

To gain insight into the vanadium redox evolution in the bulk of Na_*x*_V_2_O_5_ particles during electrochemical cycling, X-ray absorption spectroscopy (XAS) data have been collected at the V K-edge in transmission mode for a series of Na_*x*_V_2_O_5_ electrodes recovered at different states of discharge and charge in the first cycle (Figure 5A). Normalized XAS spectra collected at the V K-edge (5465 eV), along with the various vanadium oxide standard references, are plotted in Figure 5 and Figure S9. The formal oxidation state of V is inferred from the half-height energy position of the absorption edge. The vanadium species in the bulk of the pristine ζ-V_2_O_5_ particles are present in mixed V^5+^/V^4+^ formal oxidation states due to residual Ag ions remaining after the leaching process. Upon electrochemical sodiation of Na_*x*_V_2_O_5_ to 2.0 V versus Na/Na^+^, a clear shift of around 1.7 eV of the edge position toward lower energies is observed, indicating vanadium reduction, as expected upon Na-ion insertion (Figure 5B). Further electrochemical sodiation leads to subsequent vanadium reduction to tetravalent vanadium at 1.7 V, and even beyond a formal tetravalent state at 1.0 V (Figure 5B). At the fully sodiated state (1.0 V), vanadium centers are inferred to be in mixed V^3+^/V^4+^ formal oxidation states. Upon desodiation (charging) to 4.0 V, the V K-edge XANES spectrum exhibits a shift of around 0.6 eV toward higher energy values, indicating the reoxidation of vanadium to slightly above formally tetravalent states (Figure 5C). The XANES spectra demonstrate that vanadium redox centers in the bulk of the insertion host participate in compensating for Na-ion insertion/deinsertion through reversible V^3+^/V^4+^ and V^4+^/V^5+^ redox couples. However, it is worth noting that the formal oxidation state of vanadium in the fully charged state after one cycle (sodiation to either 1.0 or 1.5 V) is lower than that of vanadium in the pristine ζ-V_2_O_5_ (∼3.1 V). In concurrence with the *operando* XRD data plotted in Figure 4, this is consistent with irreversible sodiation and stabilization of a pillared preinserted β-Na_*x*_V_2_O_5_ (*x* ∼ 0.33) phase upon discharging to 1.5 V and subsequent charging back to 4.0 V. Such pillaring facilitates subsequent reversible Na-ion insertion/deinsertion.

**Figure 5:**
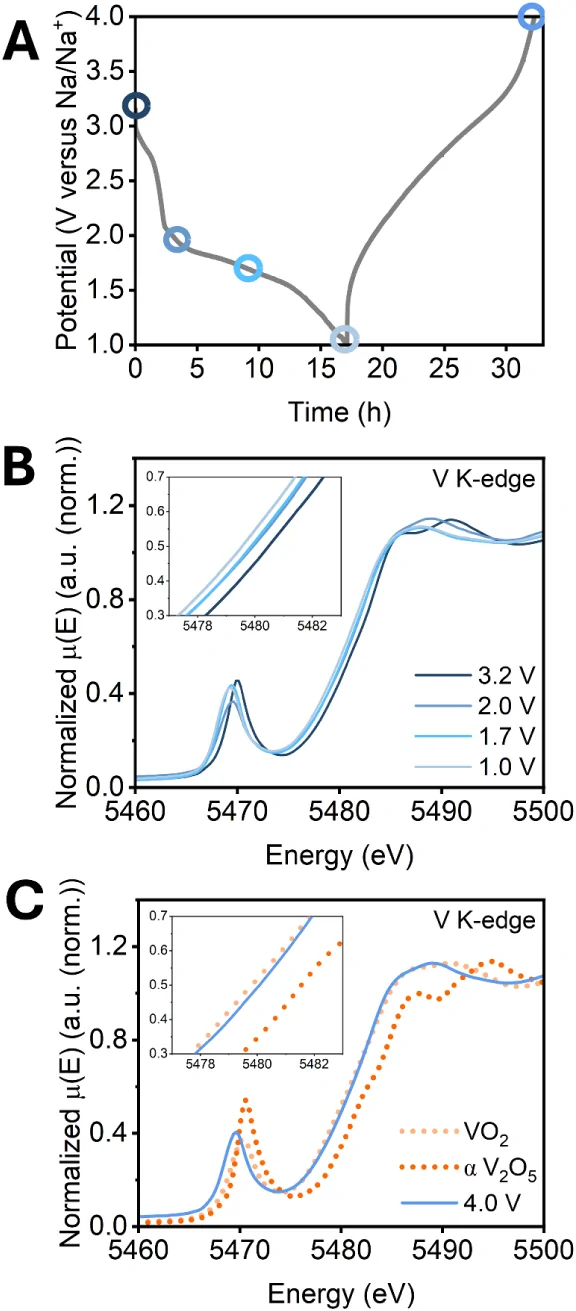
Charge compensation mechanism inferred from V K-edge XANES spectroscopy. (a) Galvanostatic curves of the first cycle of electrochemically sodiated and desodiated ζ-V_2_O_5_ at the current rate of 0.05 C in the voltage range of 1.0–4.0 V in the sodium half-cell. The points denote different discharge and charge states selected for *ex situ* XAS analysis. (b) Normalized vanadium K-edge XANES spectra of pristine ζ-V_2_O_5_, and β-Na*_x_* V_2_O_5_ electrodes at different states of charge (2.0, 1.7, and 1.0 V) and (c) a β-Na*_x_* V_2_O_5_ electrode at the fully charged state (4.0 V) after the first cycle in the voltage range of 1.0–4.0 V compared to α-V_2_O_5_, VO_2_, and V_2_O_3_ standards.

### 3.4 Sodiation Heterogeneities

The morphology of pristine ζ-V_2_O_5_ and β-Na_*x*_V_2_O_5_ at different states of charge was examined using SEM coupled with EDX analysis. Figure S10 shows SEM images of as-prepared and electrochemically sodiated β-Na_*x*_V_2_O_5_ nanowires with average dimensions of approximately 250 nm in width and ca. 2 μm in length. The morphology of the electrode particles was further examined at the fully discharged state (1.5 V) after one cycle (Figure S10B) and after 50 cycles (Figure S10D). The nanowire morphology remains largely preserved even after prolonged cycling within the voltage range of 1.5—3.5 V vs Na/Na^+^ (Figure S10D). In contrast, prolonged cycling within the extended voltage range of 1.0—3.5 V vs Na/Na^+^ results in noticeable morphological changes in β-Na_*x*_V_2_O_5_, as shown in Figure S10E. EDX analysis confirms the presence of sodium after the first discharge process to 1.5 V (Figures S11 and S12).

To visualize potential phase inhomogeneities, scanning transmission X-ray microscopy (STXM) was used to characterize samples with various sodium concentrations recovered during the first discharge process to 2.0, 1.7, and 1.5 V. Hyperspectral XAS spectra acquired at the V L-edge and O K-edge clearly evidence Na-ion insertion. First, at the V L_III_ edge there is a notable diminution of the feature at ca. 516 eV ascribed to transitions to V 3d_*xy*_ states hybridized with O 2*p* states upon Na-ion insertion, as shown in Figure 6. The diminution of this feature corresponds to Pauli blocking, as vanadium states at the conduction band edge are reduced to compensate for Na-ion insertion, as also observed by V K-edge XANES (Figure 5B). Furthermore, there is an inversion of the t_2g_ (∼530 eV)/e_g_* (∼532 eV) intensity ratios at the O K-edge, reflecting the reduction of O 2*p* – V 3*d* hybrid states and loss of degeneracy of spin-up and spin-down states. Both soft X-ray spectral features show clear signs of added electron density into the respective orbitals as a result of Na-ion insertion, corroborating the vanadium reduction inferred from V K-edge XANES (Figure 5). Additionally, hyperspectral maps generated using the reference spectra from the STXM image stack by singular value decomposition show fairly homogeneous sodiation through the bulk of the nanowires, with some edge heterogeneities likely also ascribed to the formation of a Na-rich CEI associated with some abstraction of surface oxygen and surface degradation.

**Figure 6:**
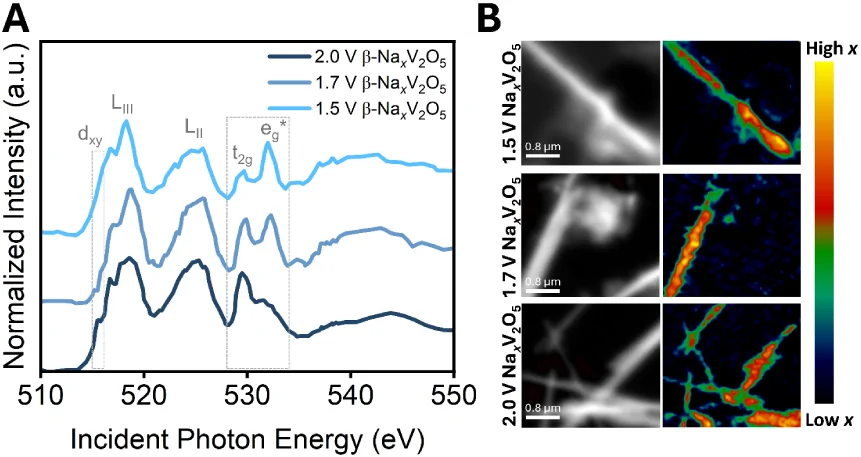
Sodiation heterogeneities mapped by STXM. (a) Average XAS spectra acquired at V L-edge and O K-edge for ζ-V_2_O_5_ nanowires electrochemically discharged to 2.0, 1.7, and 1.5 V. (b) SXTM map of nanowires (left) and composition maps (right) illustrating homogeneous sodiation through the bulk of the particles with some CEI formation along edges.

## 4 Conclusions

In this work, we have examined the insertion and migration of Na ions in a metastable V_2_O_5_ polymorph and inferred the sequence of interstitial site filling, examined ion dynamics and site-to-site migration barriers, evaluated ion-insertion-induced structural distortions and their reversibility, and deciphered charge compensation mechanisms. The interstitial sites occupied by Na ions (*x* < 0.27 in Na_*x*_V_2_O_5_) are inferred in low sodiation regimes using high-resolution single-crystal X-ray diffraction. Na ions initially occupy a seven-coordinated β-site with split-site disorder along the walls of the tunnel framework, with only subtle alterations of the tunnel and unit cell dimensions. In contrast to direct synthesis routes that position Na ions in equilibrium sites, topochemical sodiation yields a more nuanced view characterized by disordered Na ions reflecting the connectivity between interstitial sites. Combined powder X-ray and neutron diffraction analyses confirm preservation of the tunnel framework and illuminate the role of vanadyl oxygen atoms from [VO_5_] square pyramids in defining the interstitial sites where Na ions are ensconced.

Electrochemical sodiation in sodium half-cells is required to access higher sodiation stoichiometries beyond the chemical insertion limit. *Operando* powder X-ray diffraction provides insight into Na-ion site occupancies and structural evolution upon sodium insertion and extraction. XRD patterns reveal that, upon sodiation beyond *x* = 0.33, two distinct Na-ion sites (β and β′; the latter enforcing 5-fold coordination of Na ions) coexist within the tunnel structure. At higher sodium contents (*x* > 0.66 in Na_*x*_V_2_O_5_), Rietveld refinements to powder XRD patterns indicate that Na ions additionally occupy 2-fold coordinated C sites, accompanied by Na-ion redistribution and reduction of the symmetry. Upon charging, the tunnel structure remains largely intact, with Na-ion redistribution from the β′/C sites back to the β/β′ sites and restoration of the unit-cell parameters. However, electrochemical measurements, together with *operando* XRD and V K-edge XAS, indicate that approximately 0.3 Na ions per formula unit remain inserted after the first cycle and are not readily extractable upon electrochemical desodiation. The Na ions trapped in the crystal structure at the fully discharged state act as pillars that stabilize the tunnel framework, thereby enabling reversible electrochemical cycling in subsequent cycles.

The ability to reversibly insert both Li ions and Na ions in ζ-V_2_O_5_ provides the rare opportunity to contrast ion-insertion and migration mechanisms. Distinctive differences are observed in the thermodynamics and kinetics of lithiation and sodiation mechanisms in the same ζ-V_2_O_5_ polymorph insertion host. While ζ-V_2_O_5_ sustains fully reversible Li-ion insertion up to a complement of 1.91 Li ions per formula unit, the tunnel frameworks accommodate a somewhat lower concentration of Na ions up to a maximum insertion stoichiometry of 1.2 Li ions per V_2_O_5_ formula unit. Furthermore, the coordination environments within the sites are similar to those of Li ions, going from 7 → 5 → 2 when going from β-to-β′-to-C sites.^18,70^ Distinctively, ca. 0.3 Na ions per formula unit are consumed first to stabilize a preinserted β-Na_0.3_V_2_O_5_ phase, which represents a thermodynamic minimum with no clear analogues in the Li—V—O phase diagram. The pillaring influence of these Na ions enables subsequent reversible Na-ion insertion and extraction. Furthermore, the larger size of Na ions induces substantially greater structure distortions; contrasting similar extents of ion insertion, for *x* ∼ 0.33, Li_*x*_V_2_O_5_ is expanded by 0.5% with a change of β angle of <0.1% with respect to the parent ζ-V_2_O_5_ insertion host, while at higher Li concentrations (*x* ∼ 1) the β angle changes by only 1.4% and the structure preserves the same *C*2/*m* symmetry. In contrast, for the same concentration of Na ions, Na_*x*_V_2_O_5_ is expanded by 0.9% (*x* ∼ 0.33) and has a change in the β angle of 0.3%, while at higher Na concentrations, the severe structural distortion engenders a drastic change in the β angle of 3% (*x* ∼ 1) and a reduction of symmetry to the *P*2/*m* space group. Despite the substantively intensified structural distortion observed upon Na-ion insertion, the tunnel-structured framework is fully recovered upon desodiation. STXM and electrochemical cycling results suggest a greater propensity for interphase formation for Na ions as compared to Li ions, which amplifies the need to mitigate surface degradation, such as through particle coating development. Although Na and Li ions exhibit markedly different electrochemical kinetics, their mobilities remain relatively similar, differing by only 1–2 orders of magnitude depending on the state of discharge.

Overall, this work provides a comprehensive structural picture of Na-ion insertion in ζ-V_2_O_5_ across topochemical, chemical, and electrochemical methods. The results demonstrate the remarkable structural robustness of the ζ-V_2_O_5_ tunnel framework after the first sodiation process and provide new insight into the relationship between Na-site occupancy, Na-ion mobility, and structural stability in tunnel-type cathodes for Na-ion as well as dual-ion batteries. Future work will examine strategies for mitigating surface degradation, such as through coating design and site-selective modification to improve Na-ion migration kinetics.

## Supplementary Material







## Notes

Synchrotron powder X-ray diffraction and neutron powder diffraction Rietveld refinement results; single-crystal X-ray diffraction structure determination of β-Na_0.27_V_2_O_5_, including crystallographic parameters, atomic coordinates, displacement parameters, bond lengths, and bond angles; reciprocal-space reconstructions; scanning electron microscopy (SEM) images; electrochemical cycling data; V K-edge XANES spectra; SEM-EDX elemental mapping and spectra of pristine and sodiated electrodes (PDF)
Crystallographic Information File (CIF) for β-Na_0.27_V_2_O_5_ containing refined atomic coordinates, occupancies, displacement parameters, bond lengths, bond angles, and crystallographic parameters obtained from single-crystal X-ray diffraction (CIF)
checkCIF/PLATON report (PDF)

## Notes

Deposition Number 2566400 contains the supplementary crystallographic data for this paper. These data can be obtained free of charge via the joint Cambridge Crystallographic Data Centre (CCDC) and Fachinformationszentrum Karlsruhe Access-Structures-service.

## Abbreviations

SHE: Standard Hydrogen Electrode
CEI: Cathode Electrolyte Interphase
