# Supplementary material for: Na-Ion Interstitial Site Filling and Structural Evolution in a Tunnel-Structured Vanadium Oxide Insertion Host
Source: ACS Mater Au. 2026 Sep 30;6(6):1659–72. doi: 10.1021/acsmaterialsau.6c00206 (PMC13760625; doi:10.1021/acsmaterialsau.6c00206)
Supplement: Supplementary file 1 [file mg6c00206_si_001.pdf]

Na-ion interstitial site filling and structural evolution in a tunnel-structured vanadium oxide insertion host

James Pérez-Vázquez, [a,b] Sarbajeet Chakraborty, [a,b] Yu-Hsiang Chiang, [a,b] Aysan A. Yilmaz, [a,b] Victor A. Gomez, [a,b] Yuting Luo, [c] Leonardo Gobbato, [a,b] David A. Santos, [d] Cade Alaniz,[b,d] Denis Sheptyakov, [e] Grigory Smolentsev, [f] Olga Safonova, [f] Maarten Nachtegaal, [f] Mohammed Al-Hashimi, [g] Sarbajit Banerjee, [a,b] Diana Quintero-Castro, [e*] Dominika Baster, [b*]

[a] Laboratory for Inorganic Chemistry, Department of Chemistry and Applied Biosciences, ETH Zürich, Vladimir-Prelog-Weg 2, 8093 Zürich, Switzerland

[b] PSI Center for Energy and Environmental Sciences, Paul Scherrer Institute, Forschungsstrasse 111, 5232 Villigen PSI, Switzerland

[c] Department of Materials Science and Engineering, Johns Hopkins University, Baltimore, Maryland 21218, USA

[d] Department of Chemistry, Texas A&M University, College Station, TX 77840, USA

[e] Laboratory for Neutron Scattering and Imaging, PSI Center for Neutron and Muon Sciences, Paul Scherrer Institute, 5232 Villigen PSI, Switzerland

[f] Swiss Light Source, Paul Scherrer Institute, Forschungsstrasse 111, 5232 Villigen PSI, Switzerland

[g] College of Science and Engineering, Hamad Bin Khalifa University, P.O. Box: 34110, Doha, Qatar

Supplementary Table 1 ---------- 3

Supplementary Table 2 ---------- 5

Supplementary Table 3 ---------- 6

Supplementary Table 4 ---------- 8

Supplementary Table 5 ---------- 9

Supplementary Table 6 ---------- 10

Supplementary Table 7 ---------- 11

Supplementary Table 8 ---------- 13

Supplementary Table 9 ---------- 14

Supplementary Table 10 ---------- 15

Supplementary Figure 1 ---------- 16

Supplementary Figure 2 ---------- 16

Supplementary Figure 3 --- 17
Supplementary Figure 4 --- 17
Supplementary Figure 5 --- 18
Supplementary Figure 6 --- 18
Supplementary Figure 7 --- 19
Supplementary Figure 8 --- 20
Supplementary Figure 9 --- 21
Supplementary Figure 10 -- 22
Supplementary Figure 11 -- 22
Supplementary Figure 12 -- 23
References--- 23

Table S1. Comparison of the reported crystal structures of β-$Na_{0.33}V_2O_5$ synthesized using various synthetic approaches.

No.	Space group	Composition (*x* in monoclinic $Na_xV_2O_5$)	Method of preparation	Interstitial sites occupied	Crystal structure/ Morphology	Lattice parameters (Å), β(°)	Reference
1	*A*2/*m*	0.33	Hydrothermal with NaOH	β	Polycrystalline/ Nanowires	-	1
2	*C*2/*m*	0.33	Sol-gel ion exchange with Na_2SO_4	β	Polycrystalline Blocks	*a* = 15.4359(6) *b* = 3.6005(1) *c* = 10.0933(3) *β* = 109.570 (2)	2
3	*C*2/*m*	0.282	Hydrothermal with $NaNO_3$	β	Polycrystalline/ Nanorods	*a* = 15.4054(5) *b* = 3.6095(1) *c* = 10.0788(3) *β* = 109.546(2)	3
4	*C*2/*m*	0.35	Dehydration of layered δ phase	β	Polycrystalline/ Nanorods & prisms	*a* = 15.4039(7) *b* = 3.6074(2) *c* = 10.0687(5) *β* = 109.540 (3)	4
5	*A*2/*m* *	0.33	Sol-gel ion exchange with NaCl	β	Polycrystalline/ Platelets	*a* = 10.10(5) *b* = 3.63(1) *c* = 15.45(5) *β* = 110.0(5)	5
6	*A*2/*m*	0.33	Hydrothermal with $NaHCO_3$ and polyvinylpyrrolidone	β	Polycrystalline/ Nanosheets	*a* = 10.08 *b* = 3.61 *c* = 15.44 *β* = 109.60	6
7	*C*2/*m*	0.33	Solid-state direct synthesis	β	Monocrystalline	-	7
8	*C*2/*m*	0.32	Soft-chemistry synthesis	β	Monocrystalline	*a* = 15.3629(7) *b* = 3.6109(2) *c* = 10.0502(5) *β* = 109.5900(10)	8

9	*C*2/*m*	0.25	Sol-gel ion exchange with $NaNO_3$	β	Polycrystalline	*a* = 15.608(8) *b* = 3.609(1) *c* = 10.077(9) *β* = 109.541	8
10	*C*2/*m*	0.27	Topochemical insertion into ζ-V_2O_5 with NaI	β	Monocrystalline	*a* = 15.3488(5) *b* = 3.61050(10) *c* = 10.0803(3) β = 109.874(3)	This work
11	*C*2/*m*	0.32	Hydrothermal with $NaOOCCH_3$	β	Nanowires	*a* = 15.4131(19) *b* = 3.610122(83) *c* = 10.0708(10) β = 109.5444(35)	This work
12	*C*2/*m*	0.66	Electrochemical insertion in Na half-cell	β & β'	Polycrystalline/ Nanowires	*a* = 15.138 *b* = 3.609 *c* = 10.283 β = 107.284	This work
13	*C*2/*m*	0.70	Electrochemical insertion in Na half-cell	β & β'	Polycrystalline/ Nanowires	*a* = 15.461 *b* = 3.619 *c* = 10.255 β = 111.127	This work
14	*P*2/*m*	1.0	Electrochemical insertion in Na half-cell	β' & C	Polycrystalline/ Nanowires	*a* = 17.597 *b* = 3.659 *c* = 10.433 β = 113.448	This work

*Inferred from source

Table S2. Parameters for Rietveld refinements to X-ray powder diffraction performed for ζ-V_2O_5 (β-$Ag_{0.067}V_2O_5$).

ζ-V_2O_5 (β-$Ag_{0.067}V_2O_5$) XRD Rietveld refinement statistics						
wR	5.53%	R	3.94%	χ^2	1.21	

2θ (°)	0.89-13.5	Radiation	Synchrotron 0.16520 Å	Temp	295 K	
Space group:	*C*2/*m*	V(Å³)	523.301(43)	Z	6	
a (Å)	15.2934(39)	*b* (Å)	3.60719(17)	*c* (Å)	10.0849(22)	
α(°)	90	β(°)	109.8470(73)	γ(°)	90	
atom	*x*	*y*	*z*	frac	Uiso	Wyckoff
Ag	0.9883(32)	0	0.4007(46)	0.090	0.02214	4i
V1	0.11709(50)	0	0.11478(79)	1	0.00081	4i
V2	0.33930(54)	0	0.10406(84)	1	0.00784	4i
V3	0.28985(55)	0	0.41146(90)	1	0.00265	4i
O1	0	0	0	1	0.03941	2a
O2	0.1106(20)	0	0.2710(26)	1	0.00820	4i
O3	0.13289(23)	0.5000	0.0770(28)	1	0.01057	4i
O4	0.2672(18)	0	0.2234(29)	1	0.00245	4i
O5	0.4374(17)	0	0.2211(26)	1	0.00108	4i
O6	0.3183(18)	0.5	0.0548(29)	1	0.00588	4i
O7	0.3964(17)	0	0.4773(29)	1	0.00058	4i
O8	0.2649(23)	0.5	0.4324(33)	1	0.03003	4i

Table S3. Crystal data and structure refinement for structure solution of b-$Na_{0.27}V_2O_5$ from single crystal X-ray diffraction.

**Identification code**	zvo+NaI
**Empirical formula**	$Na_{0.27}V_2O_5$
**Formula weight**	564.26

**Temperature/K**	100.00(10)	
**Crystal system**	monoclinic	
**Space group**	*C2/m*	
**Unit Cell Dimensions**	*a*=15.3488(5) Å<br>*b*=3.61050(10) Å<br>*c*=10.0803(3) Å	α=90°<br>β=109.874(3)°<br>γ=90°
**Volume/Å^3**	525.35(3)	
**Z**	6	
**ρ_{calc}g/cm^3**	3.567	
**μ/mm^{-1}**	5.247	
**F (000)**	534.0	
**Crystal size/mm^3**	0.26 × 0.12 × 0.08	
**Radiation**	Mo K_α (λ = 0.71073)	
**2θ range for data collection/°**	4.296 to 71.62	
**Diffractometer**	Rigaku Saturn724+ (2x2 bin mode)	
**Absorption correction**	Gaussian *CrysAlis PRO* 1.171.43.90 (Rigaku Oxford Diffraction, 2023). Numerical absorption correction based on Gaussian integration over a multifaceted crystal model. Empirical absorption correction using spherical harmonics, implemented in SCALE3 ABSPACK scaling algorithm.	
**Index ranges**	-24 ≤ h ≤ 24, -5 ≤ k ≤ 5, -16 ≤ l ≤ 16	
**Reflections collected**	11078	
**Independent reflections**	1307 [R_{int} = 0.0279, R_{sigma} = 0.0138]	
**Data/restraints/parameters**	1307/0/79	
**Goodness-of-fit on F^2**	1.242	
**Final R indexes [I>=2σ (I)]**	R_1 = 0.0210, wR_2 = 0.0532	
**Final R indexes [all data]**	R_1 = 0.0216, wR_2 = 0.0535	

Largest diff. peak/hole / e Å^{-3}	0.78/-0.57

Table S4. Fractional Atomic Coordinates (×10^{-4}) and Equivalent Isotropic Displacement Parameters (Å^2×10^{-3}) for b-$Na_{0.27}V_2O_5$. U_{eq} is defined as 1/3 of the trace of the orthogonalized U_{iJ} tensor.

Atom	*x*	*y*	*z*	Occupancy	U(eq)
V1	7111.3(2)	10000	886.7(3)	1	5.87(7)
V2	6620.0(2)	10000	3985.3(3)	1	5.56(7)
V3	6175.4(2)	5000	6187.9(3)	1	6.59(7)
O1	6006.4(11)	10000	258.1(16)	1	11.2(3)

Atom	*x*	*y*	*z*	Occupancy	U(eq)
O2	7426.4(11)	5000	739.8(15)	1	7.8(2)
O3	7350.4(10)	10000	2761.0(15)	1	6.9(2)
O4	5616.3(11)	10000	2811.8(16)	1	10.7(3)
O5	6852.4(10)	5000	4456.5(15)	1	7.8(2)
O6	5000	5000	5000	1	9.0(3)
O7	6346.4(10)	10000	5777.8(15)	1	6.5(2)
O8	6085.4(11)	5000	7730.3(16)	1	9.4(2)
Na1	5018(4)	5000	9040(30)	0.270	9(2)
Na2	4995(7)	5000	8560(50)	0.130	10(4)

Table S5. Anisotropic Displacement Parameters ($Å^2×10^3$) for b-$Na_{0.27}V_2O_5$. The anisotropic displacement factor exponent takes the form: $-2π^2[h^2a^{*2}U_{11}+2hka^*b^*U_{12}+…]$.

Atom	U_{11}	U_{22}	U_{33}	U_{23}	U_{13}	U_{12}
V1	9.26(13)	3.47(13)	5.81(12)	0	3.77(10)	0
V2	7.97(13)	3.44(13)	5.69(12)	0	2.86(10)	0
V3	10.71(14)	3.14(13)	7.12(13)	0	4.59(11)	0
O1	10.8(6)	14.2(7)	8.6(6)	0	3.3(5)	0
O2	14.3(6)	3.7(5)	7.1(5)	0	6.0(5)	0

Atom	U_{11}	U_{22}	U_{33}	U_{23}	U_{13}	U_{12}
O3	10.2(6)	5.0(5)	6.0(5)	0	3.5(4)	0
O4	10.8(6)	12.1(7)	8.6(6)	0	2.6(5)	0
O5	11.2(6)	4.1(5)	8.5(6)	0	4.0(5)	0
O6	9.1(8)	5.0(8)	12.0(8)	0	2.4(7)	0
O7	10.2(6)	3.9(5)	5.8(5)	0	3.4(4)	0
O8	13.6(6)	7.7(6)	7.3(5)	0	4.0(5)	0
Na1	9.8(16)	10.8(16)	8(6)	0	3.6(18)	0
Na2	10(3)	12(3)	7(11)	0	3(4)	0

Table S6. Bond Lengths [Å] and Bond Angles [˚] for b-$Na_{0.27}V_2O_5$.

Atom Pairs	Bond Length (Å)	Atom Pairs	Bond Length (Å)	Atom Pairs	Bond Length (Å)
V 1—V1^{1}	3.0542(5)	V2—O5^{3}	1.8699(4)	O1—Na2^{8}	2.88(3)
V1—V1^{2}	3.0542(5)	V2—O4	1.5917(16)	O1—Na2^{7}	2.88(3)
V1—O3	1.7980(14)	V2—Na2^{7}	3.42(3)	O1—Na2^{5}	2.61(3)
V1—O2	1.8877(4)	V2—Na2^{8}	3.42(3)	O1—Na2^{4}	2.61(3)
V1—O2^{3}	1.8877(4)	V3—O3^{6}	2.1477(15)	O8—Na1	2.427(19)
V1—O2^{2}	1.9955(14)	V3—O7^{9}	1.8901(4)	O8—Na2	2.11(3)
V1—O1	1.5959(16)	V3—O7	1.8901(4)	O4—Na1^{7}	2.539(16)
V1—Na1^{4}	3.604(8)	V3—O5	2.3150(15)	O4—Na1^{8}	2.539(16)
V1—Na1^{5}	3.604(8)	V3—O8	1.6070(15)	O4—Na2^{8}	2.27(3)

V2—V3^{3}	3.1119(4)	V3—O6	1.7943(4)	O4—Na2^{7}	2.27(3)
V2—V3	3.1120(4)	V3—Na2	3.46(5)	Na1—Na1^{3}	3.61050(18)
V2—O3	1.9290(14)	O1—Na1^{4}	2.410(9)	Na1—Na1^{10}	1.96(5)
V2—O7	1.9892(14)	O1—Na1^{5}	2.410(9)	Na1—Na2^{10}	2.43(8)
V2—O5	1.8699(4)	O1—Na1^{7}	2.644(11)	Na2—Na2^{10}	2.90(11)
V2—O5^{6}	2.3368(15)	O1—Na1^{8}	2.644(11)		

^{1}3/2-X,5/2-Y,-Z; ^{2}3/2-X,3/2-Y,-Z; ^{3}+X,1+Y,+Z; ^{4}+X,+Y,-1+Z; ^{5}+X,1+Y,-1+Z; ^{6}3/2-X,3/2-Y,1-Z; ^{7}1-X,1-Y,1-Z; ^{8}1-X,2-Y,1-Z; ^{9}+X,-1+Y,+Z; ^{10}1-X,1-Y,2-Z

Table S7. Bond Lengths [Å] and Bond Angles [˚] for b-$Na_{0.27}V_2O_5$.

Atoms	Bond Angle (˚)	Atoms	Bond Angle (˚)	Atoms	Bond Angle (˚)
V1^{1}—V1—V1^{2}	72.466(14)	O3—V2—Na2^{6}	87.2(7)	O7^{9}—V3—V2	108.51(4)
V1^{2}—V1—Na1^{3}	116.8(4)	O3—V2—Na2^{7}	87.2(7)	O7—V3—V2	37.76(4)
V1^{1}—V1—Na1^{4}	116.8(4)	O7—V2—V3^{5}	35.584(6)	O7—V3—O3^{8}	84.64(5)
V1^{2}—V1—Na1^{4}	81.9(3)	O7—V2—V3	35.584(6)	O7^{9}—V3—O3^{8}	84.63(5)
V1^{1}—V1—Na1^{3}	81.9(3)	O7—V2—O5^{8}	82.12(6)	O7—V3—O7^{9}	145.53(8)
O3—V1—V1^{1}	129.36(3)	O7—V2—Na2^{7}	111.0(8)	O7^{9}—V3—O5	72.77(4)
O3—V1—V1^{2}	129.36(3)	O7—V2—Na2^{6}	111.0(7)	O7—V3—O5	72.77(4)
O3—V1—O2^{1}	149.40(7)	O5—V2—V3^{5}	116.62(5)	O7^{9}—V3—Na2	107.21(4)
O3—V1—O2^{5}	96.68(5)	O5^{8}—V2—V3^{5}	86.20(3)	O7—V3—Na2	107.20(4)
O3—V1—O2	96.67(5)	O5^{5}—V2—V3^{5}	47.77(5)	O5—V3—V2	36.735(10)

O3—V1—Na1[3]	111.8(4)	O5—V2—V3	47.77(5)	O5—V3—V2[9]	36.735(11)
O3—V1—Na1[4]	111.8(4)	O5[8]—V2—V3	86.20(3)	O5—V3—Na2	175.5(5)
O2—V1—V1[1]	39.41(4)	O5[5]—V2—V3	116.62(5)	O8—V3—V2[9]	143.331(15)
O2—V1—V1[2]	110.97(4)	O5—V2—O3	93.46(5)	O8—V3—V2	143.331(15)
O2[5]—V1—V1[2]	39.41(4)	O5[5]—V2—O3	93.46(5)	O8—V3—O3[8]	86.87(7)
O2[1]—V1—V1[1]	36.907(11)	O5[5]—V2—O7	81.39(5)	O8—V3—O7[9]	106.00(4)
O2[5]—V1—V1[1]	110.97(4)	O5—V2—O7	81.39(5)	O8—V3—O7	106.00(4)
O2[1]—V1—V1[2]	36.907(11)	O5—V2—O5[8]	76.59(5)	O8—V3—O5	159.66(7)
O2—V1—O2[1]	76.31(4)	O5[5]—V2—O5[8]	76.59(5)	O8—V3—O6	104.36(6)
O2[5]—V1—O2[1]	76.31(4)	O5[5]—V2—O5	149.78(9)	O8—V3—Na2	24.9(5)
O2—V1—O2[5]	146.00(9)	O5[8]—V2—Na2[7]	144.22(15)	O6—V3—V2	86.471(12)
O2[1]—V1—Na1[4]	94.5(4)	O5[5]—V2—Na2[7]	136.7(3)	O6—V3—V2[9]	86.471(12)
O2—V1—Na1[3]	71.80(7)	O5[5]—V2—Na2[6]	73.0(3)	O6—V3—O3[8]	168.77(4)
O2[5]—V1—Na1[4]	71.80(7)	O5[8]—V2—Na2[6]	144.22(15)	O6—V3—O7	92.12(4)
O2[5]—V1—Na1[3]	130.21(19)	O5—V2—Na2[7]	73.0(3)	O6—V3—O7[9]	92.12(5)
O2—V1—Na1[4]	130.21(19)	O5—V2—Na2[6]	136.7(3)	O6—V3—O5	95.97(4)
O2[1]—V1—Na1[3]	94.5(4)	O4—V2—V3	97.97(5)	O6—V3—Na2	79.5(5)
O1—V1—V1[2]	109.96(5)	O4—V2—V3[5]	97.97(5)	Na1[12]—Na1—V1[11]	78.9(6)
O1—V1—V1[1]	109.96(5)	O4—V2—O3	98.67(7)	Na1[12]—Na1—V1[10]	78.9(6)
O1—V1—O3	103.12(7)	O4—V2—O7	103.02(7)	Na1[12]—Na1—Na1[5]	90.001(1)
O1—V1—O2[1]	107.48(7)	O4—V2—O5[8]	174.87(7)	Na1[12]—Na1—Na2[12]	1.0(4)
O1—V1—O2	103.58(5)	O4—V2—O5[5]	103.98(5)	Na2[12]—Na1—Na1[5]	90.001(3)
O1—V1—O2[5]	103.59(5)	O4—V2—O5	103.98(5)	Na1[12]—Na2—Na2[12]	0.7(3)
O1—V1—Na1[3]	32.25(11)	O4—V2—Na2[6]	33.86(11)		
O1—V1—Na1[4]	32.25(11)	O4—V2—Na2[7]	33.86(11)		
Na1[3]—V1—Na1[4]	60.12(16)	Na2[7]—V2—Na2[6]	63.8(6)		
V3[5]—V2—V3	70.915(10)	V2—V3—V2[9]	70.914(10)		
V3—V2—Na2[6]	124.3(5)	V2—V3—Na2	141.94(17)		
V3[5]—V2—Na2[7]	124.3(5)	V2[9]—V3—Na2	141.94(17)		
V3—V2—Na2[7]	87.2(7)	O3[8]—V3—V2[9]	84.39(3)		
V3[5]—V2—Na2[6]	87.2(7)	O3[8]—V3—V2	84.39(3)		
O3—V2—V3[5]	140.588(19)	O3[8]—V3—O5	72.80(5)		
O3—V2—V3	140.588(19)	O3[8]—V3—Na2	111.8(5)		
O3—V2—O7	158.32(6)	O7—V3—V2[9]	108.51(4)		
O3—V2—O5[8]	76.20(6)	O7[9]—V3—V2[9]	37.76(4)		

[1]3/2-X,3/2-Y,-Z; [2]3/2-X,5/2-Y,-Z; [3]+X,+Y,-1+Z; [4]+X,1+Y,-1+Z; [5]+X,1+Y,+Z; [6]1-X,2-Y,1-Z; [7]1-X,1-Y,1-Z; [8]3/2-X,3/2-Y,1-Z; [9]+X,-1+Y,+Z; [10]+X,+Y,1+Z; [11]+X,-1+Y,1+Z; [12]1-X,1-Y,2-Z

Table S8. Rietveld refinement results for powder XRD patterns acquired for hydrothermally synthesized β-$Na_{0.32}V_2O_5$.

β-$Na_{0.32}V_2O_5$ XRD Rietveld refinement statistics						
wR	3.42%	R	2.43%	χ^2	0.145	
2θ (°)	0.5-7.5	Radiation	Synchrotron 0.11594 Å	Temp	295 K	
Space group:	*C*2/*m*	V(Å³)	528.084(21)	Z	6	
a (Å)	15.4131(19)	*b* (Å)	3.610122(83)	*c* (Å)	10.0708(10)	

α(°)	90	β(°)	109.5444(35)	γ(°)	90	
atom	*x*	*y*	*z*	frac	Uiso	Wyckoff
V1	0.28629(34)	1	0.90908(47)	1	0.02032	4i
V2	0.33651(30)	1	0.59993(44)	1	0.02261	4i
V3	0.38544(33)	0.5	0.38159(44)	1	0.02271	4i
O4	0.39714(81)	1	0.9751(11)	1	0.02026	4i
O5	0.25371(81)	1.5	0.9244(11)	1	0.01093	4i
O6	0.26164(80)	1	0.72800(12)	1	0.01128	4i
O7	0.43808(70)	1	0.7147(12)	1	0.02139	4i
O8	0.31273(93)	1.5	0.5503(15)	1	0.06694	4i
O9	0.5	0.5	0.5	1	0.00174	2c
O10	0.36659(90)	1	0.4230(11)	1	0.01041	4i
O11	0.39509(94)	0.5	0.2276(11)	1	0.03076	4i
Na12	0.5056(14)	0.5 08(38)	0.9044(19)	0.239	0.02931	8j

Table S9. Rietveld refinement results for neutron powder diffraction data acquired for hydrothermally synthesized β-$Na_xV_2O_5$.

β-$Na_xV_2O_5$ NPD Rietveld refinement statistics						
IneawR	5.75%	R	4.47%	χ^2	0.340	
2θ (°)	2.6-164.9	Radiation	Neutron 1.494 Å	Temp	1.5 K	
Space group:	*C*2/*m*	V(Å³)	523.988(17)	Z	6	
a (Å)	15.3448(17)	*b* (Å)	3.608431(79)	*c* (Å)	10.04479(80)	

α(°)	90	β(°)	109.5912(29)	γ(°)	90	
atom	*x*	*y*	*z*	frac	Uiso	Wyckoff
V1	0.2794(38)	1	0.8979(60)	1	0.00929	4i
V2	0.3443(33)	1	0.5729(54)	1	0.00651	4i
V3	0.3844(44)	0.5	0.3837(69)	1	0.02079	4i
O4	0.39875(37)	1	0.97401(58)	1	0.02108	4i
O5	0.25530(36)	1.5	0.92341(54)	1	0.01651	4i
O6	0.26432(32)	1	0.72466(50)	1	0.01223	4i
O7	0.43763(38)	1	0.71733(56)	1	0.01835	4i
O8	0.31469(39)	1.5	0.55604(53)	1	0.01286	4i
O9	0.5	0.5	0.5	1	0.02036	2c
O10	0.36580(34)	1	0.42231(54)	1	0.01520	4i
O11	0.39223(35)	0.5	0.22757(58)	1	0.01711	4i
Na13	0.4979(11)	0.51000	0.89622(18)	0.295	0.02930	8j

Table S10. Comparison of the electrochemical performance of monoclinic tunnel-structured V_2O_5 -based cathode materials for sodium-ion batteries (NIBs), including β-$Na_xV_2O_5$ (x_{Na} ≈ 0.33) phases prepared by various direct synthesis methods and electrochemical cycling starting from the empty ζ-V_2O_5 polymorph reported in this work.

No.	Starting material	Initial discharge capacity	Initial discharge capacity change (1 and 2 cycles)	Capacity retention	Cycling voltage window and conditions	Reference
1	β-$Na_{0.33}V_2O_5$	~85 mAh g^{-1}	~+5.9%	95%<br>50 cycles	1.5–4.0 V vs. Na/Na^+<br>20 mA g^{-1}	1
2	β-$Na_{0.282}V_2O_5$	240 mA h g^{-1} at 50 mA g^{-1}	+44%	98.33%<br>70 cycles	1.5–4.0 V vs. Na/Na^+	3
3	β-$Na_{0.33}V_2O_5$ @graphene	213 mA h g^{-1} at 20 mA g^{-1}	-	83.3% over 100 cycles	1.5–4.0 V vs. Na/Na^+<br>50 mA g^{-1}	6
4	ζ-V_2O_5	170 mAh g^{-1}	-23.5%	40% between 10-50 cycles at 0.2 C	1.5–3.5 V vs. Na/Na^+<br>0.05 C	This work
5	β-$Na_{0.33}V_2O_5$	122 mAh g^{-1}	-19.9%	-	1.5–3.5 V vs. Na/Na^+<br>0.05 C	This work

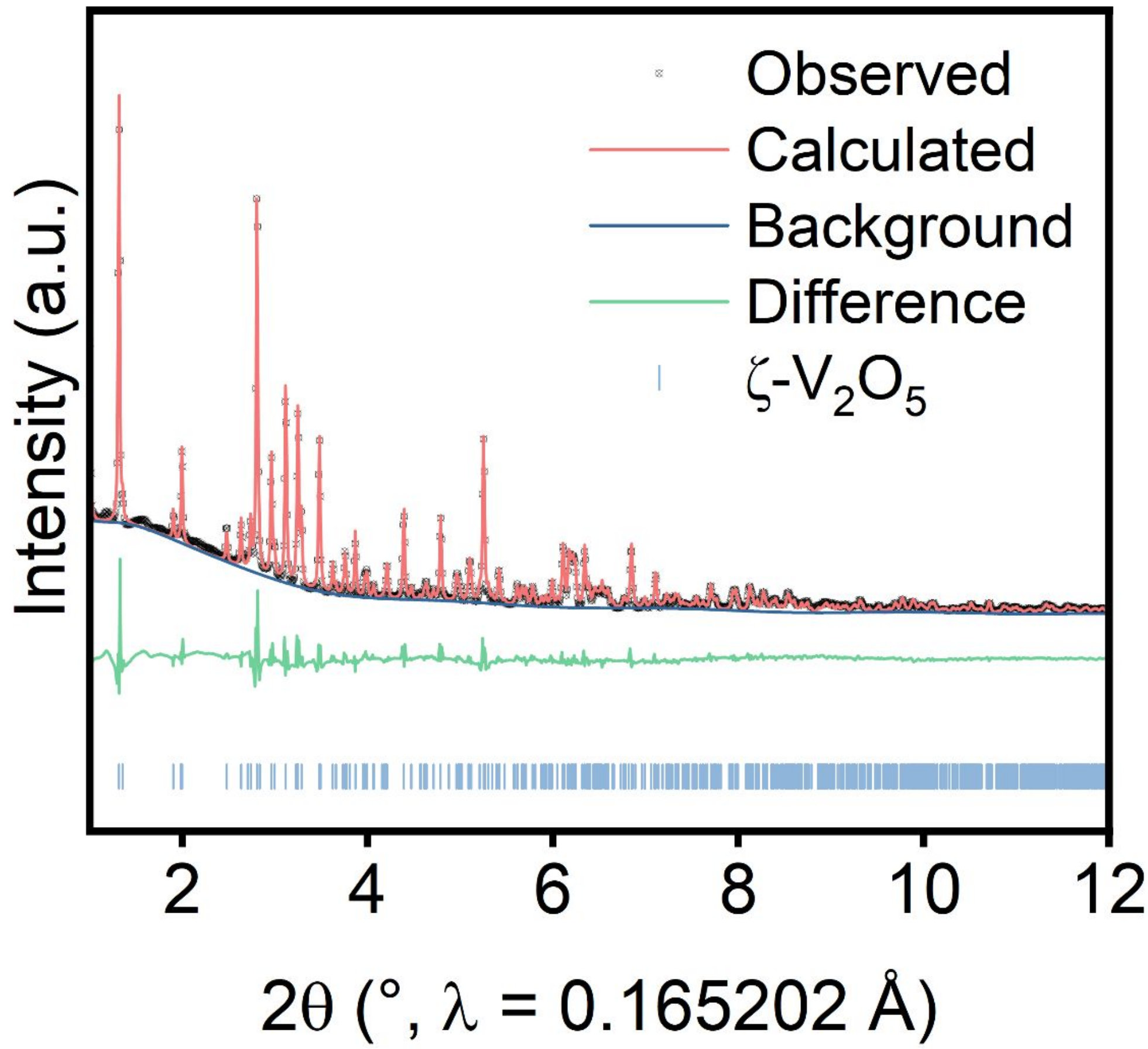

Figure S1. Rietveld refinement of powder X-ray diffraction patterns (λ = 0.165202 Å) for ζ-V_2O_5 (β-$Ag_{0.067}V_2O_5$) powder collected at room temperature. Observed, calculated, and difference profiles are shown together with the Bragg reflection positions.

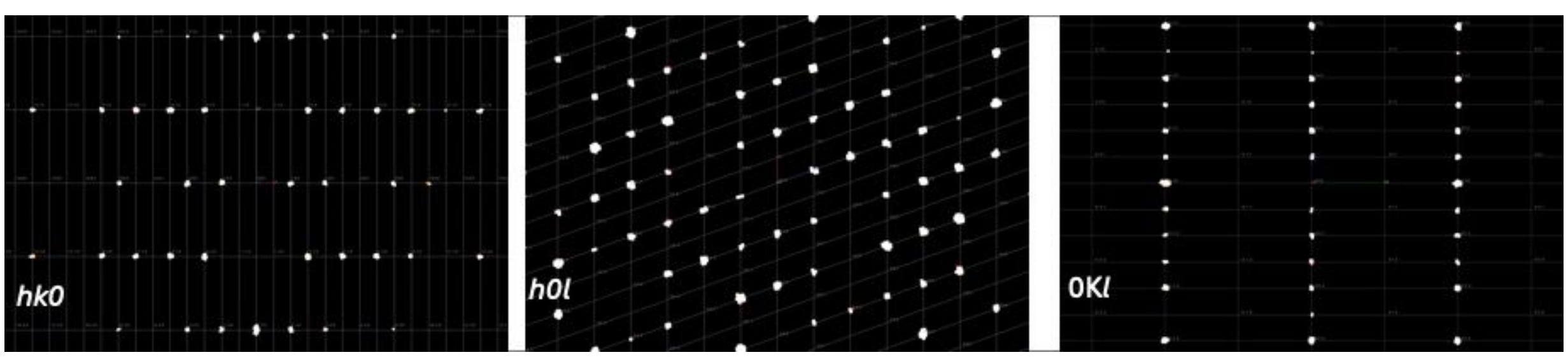

Figure S2. Crystallographic evidence of absence of long-range position correlation and Structure modulations in b-$Na_{0.27}V_2O_5$ single crystal at 100K. Reciprocal space reconstruction shows reciprocal lattice planes (*hk*0), (*h*0*l*), and (0*kl*) reconstructed from the full diffraction sphere. The sharp, well-defined Bragg reflections conform entirely to the average monoclinic *C*2/*m* lattice parameters. The complete absence of diffuse scattering, satellite reflections, or fractional interstitial intensities (e.g., k=n+1/2) between the primary Bragg peaks definitively confirms the lack of long-range zig-zag ordering or modulation q-vectors at 100 K.

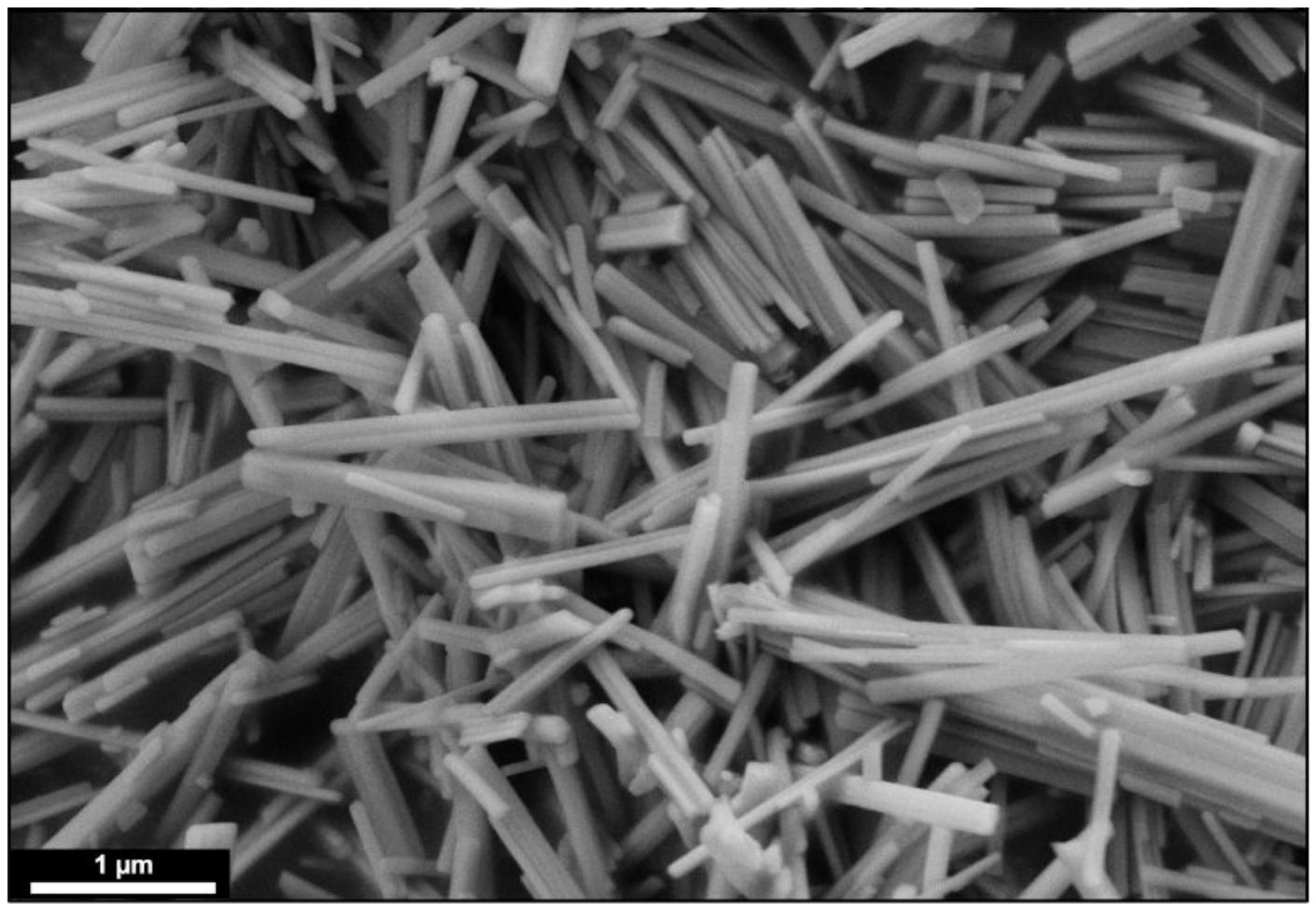

Figure S3. Scanning electron microscopy images of β-$Na_xV_2O_5$ nanowires synthesized via
a hydrothermal route, acquired at an accelerating voltage of 2 keV.

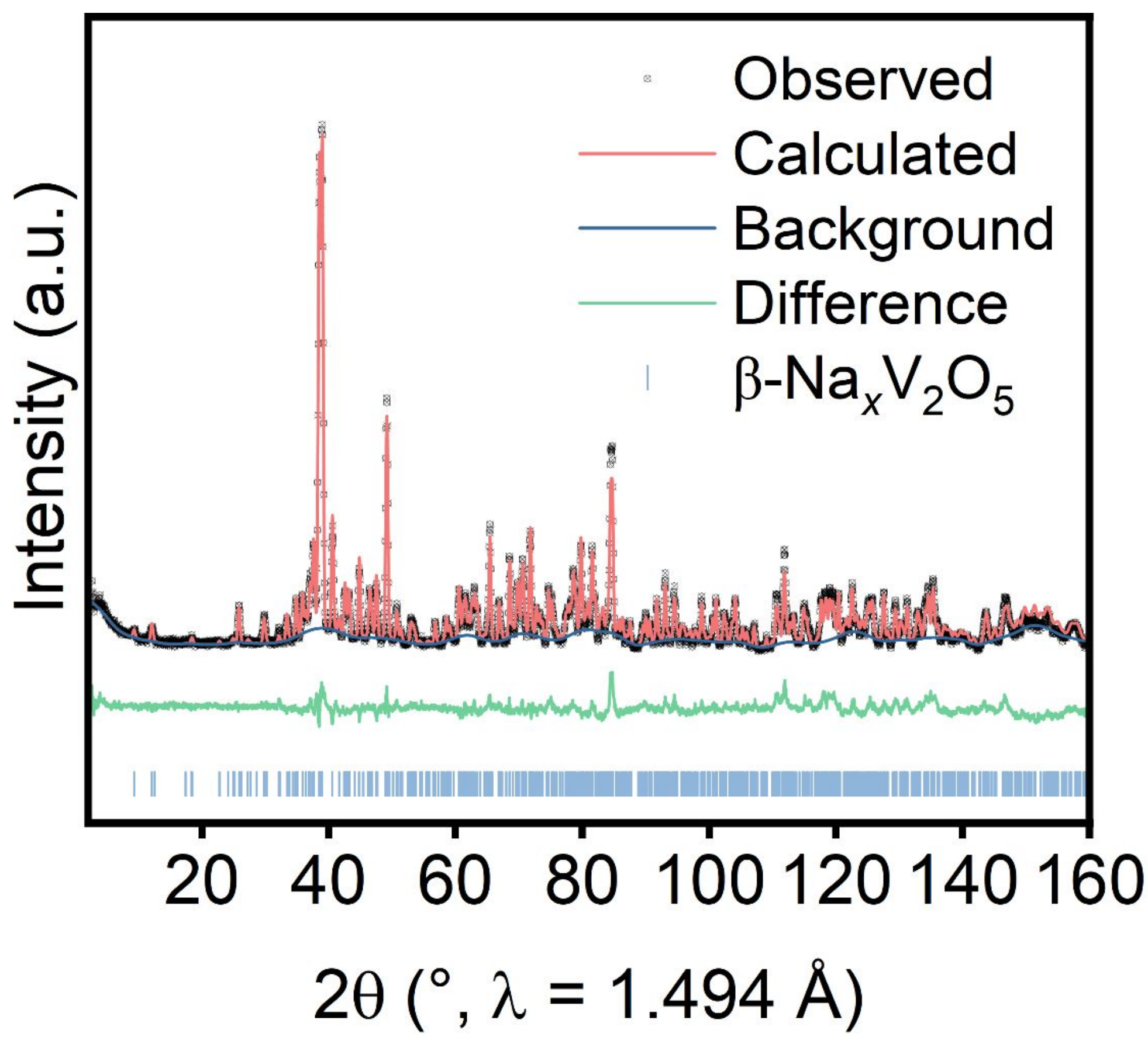

Figure S4. Rietveld refinement of neutron powder diffraction (HRPT) data (λ = 1.494 Å) for hydrothermally synthesized β-$Na_xV_2O_5$ collected at T = 1.5 K. Observed, calculated, and difference profiles are shown together with the Bragg reflection positions.

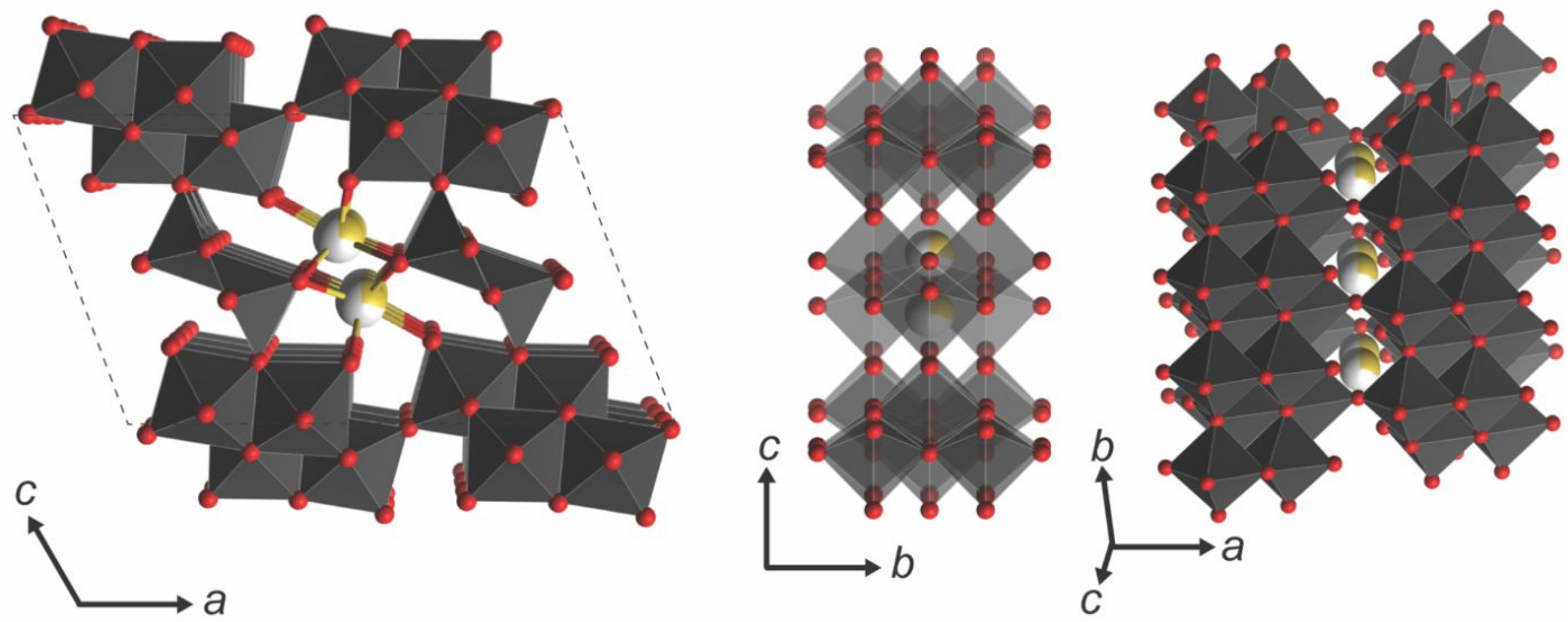

Figure S5. Crystal structure of hydrothermally synthesized β-$Na_xV_2O_5$ obtained from neutron powder diffraction Rietveld refinement (λ = 1.494 Å) shown in **Figure S4**, viewed along different crystallographic axes.

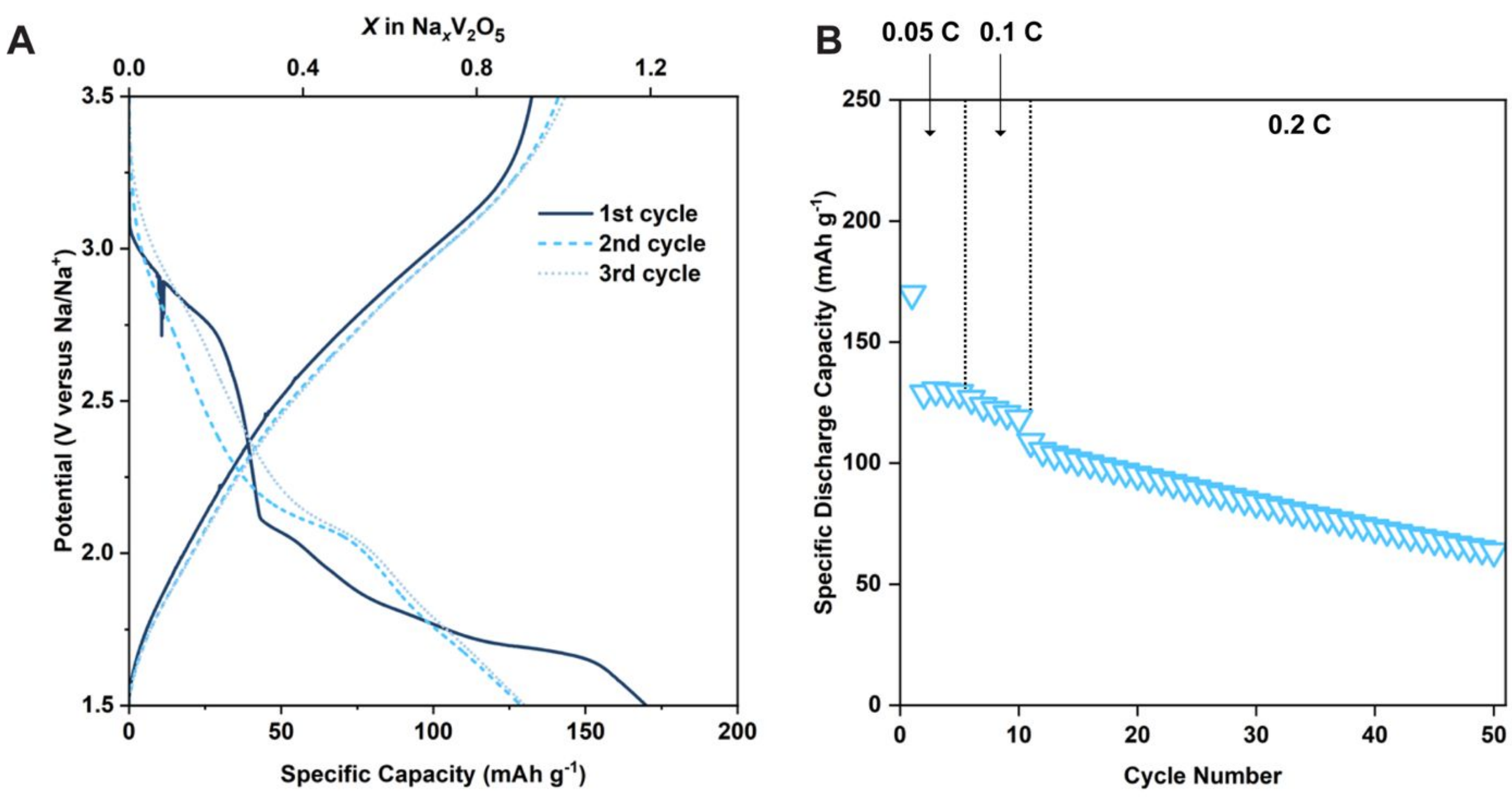

Figure S6. (a) Galvanostatic curves of ζ-V_2O_5 in the sodium half-cell at a current rate of 0.05 C in the potential range of 1.5—3.5 V. (b) Long-term cycling performance of ζ-V_2O_5 at current rates of 0.05 C, 0.1 C, and 0.2 C in the voltage range of 1.5—3.5 V.

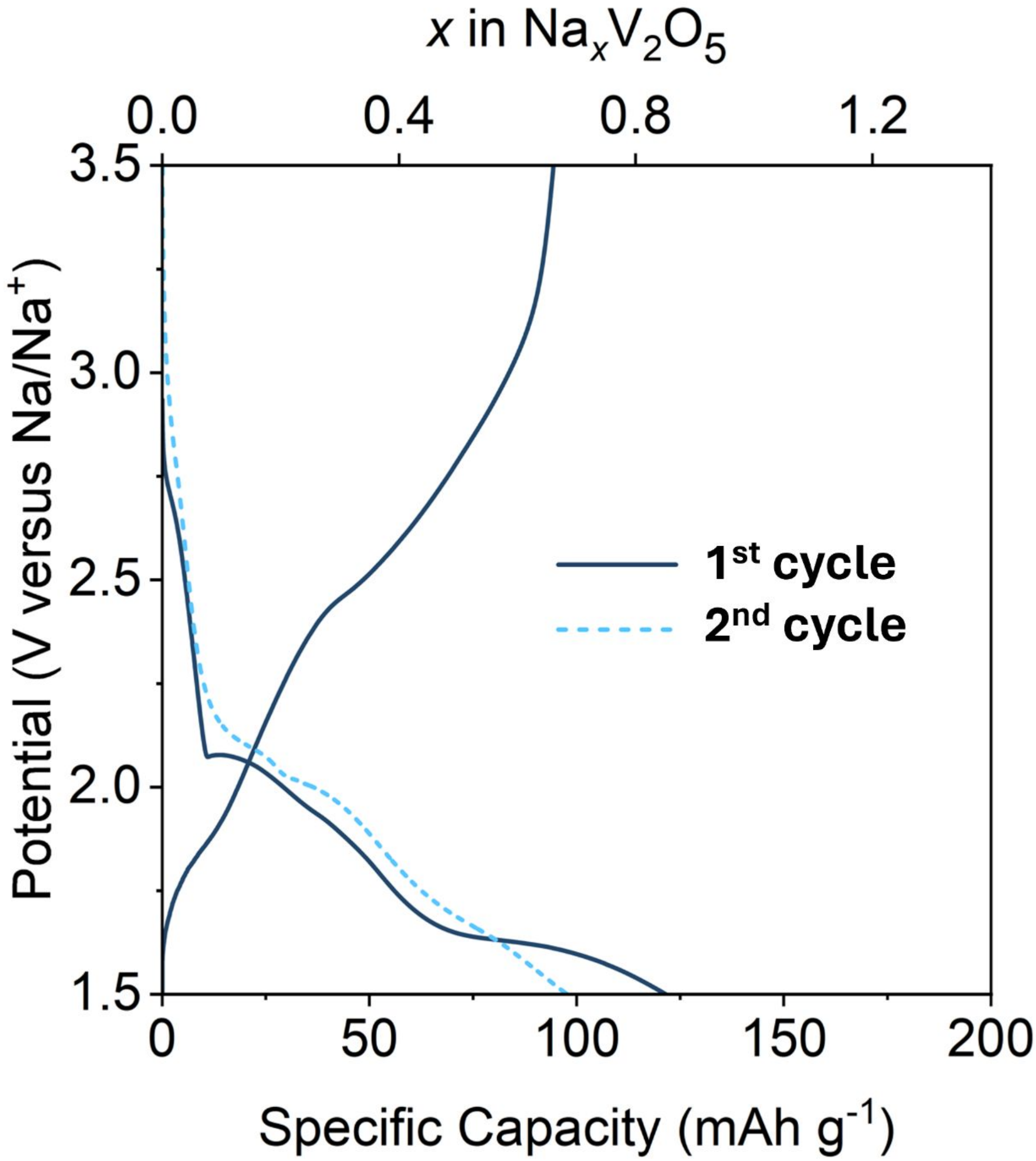

Figure S7. Galvanostatic curves of β-$Na_{0.32}V_2O_5$ obtained via a hydrothermal synthetic route, measured in a sodium half-cell at 0.05 C in the voltage range of 1.5-3.5 V (vs Na/Na^+).

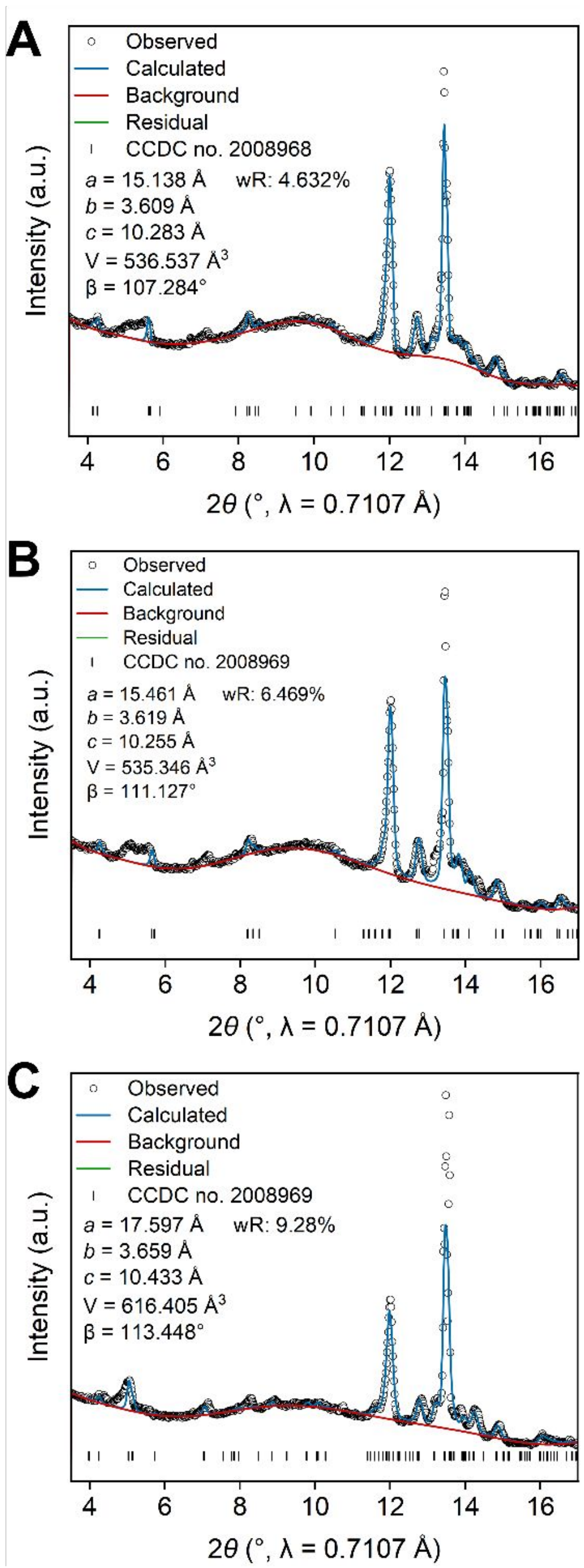

Figure S8. Rietveld refinement of *operando* X-ray diffraction patterns (λ = 0.7107 Å) collected for ζ-V_2O_5 at different stages of sodiation: (a) Regime II (x_{Na} ≈ 0.66), (b) Regime III (x_{Na} ≈ 0.70), and (c) the fully sodiated state (x_{Na} ≈ 1.0), where the crystal symmetry is further reduced to the monoclinic $P2_1$ space group. Open circles represent the experimental data, the blue lines the calculated diffraction profiles, the red lines the fitted

background, and the green lines the difference (observed minus calculated) profiles. Vertical tick marks indicate the Bragg reflection positions of the refined phases. The refined lattice parameters, unit-cell volumes, and weighted-profile reliability factors (wR) are provided for each refinement.

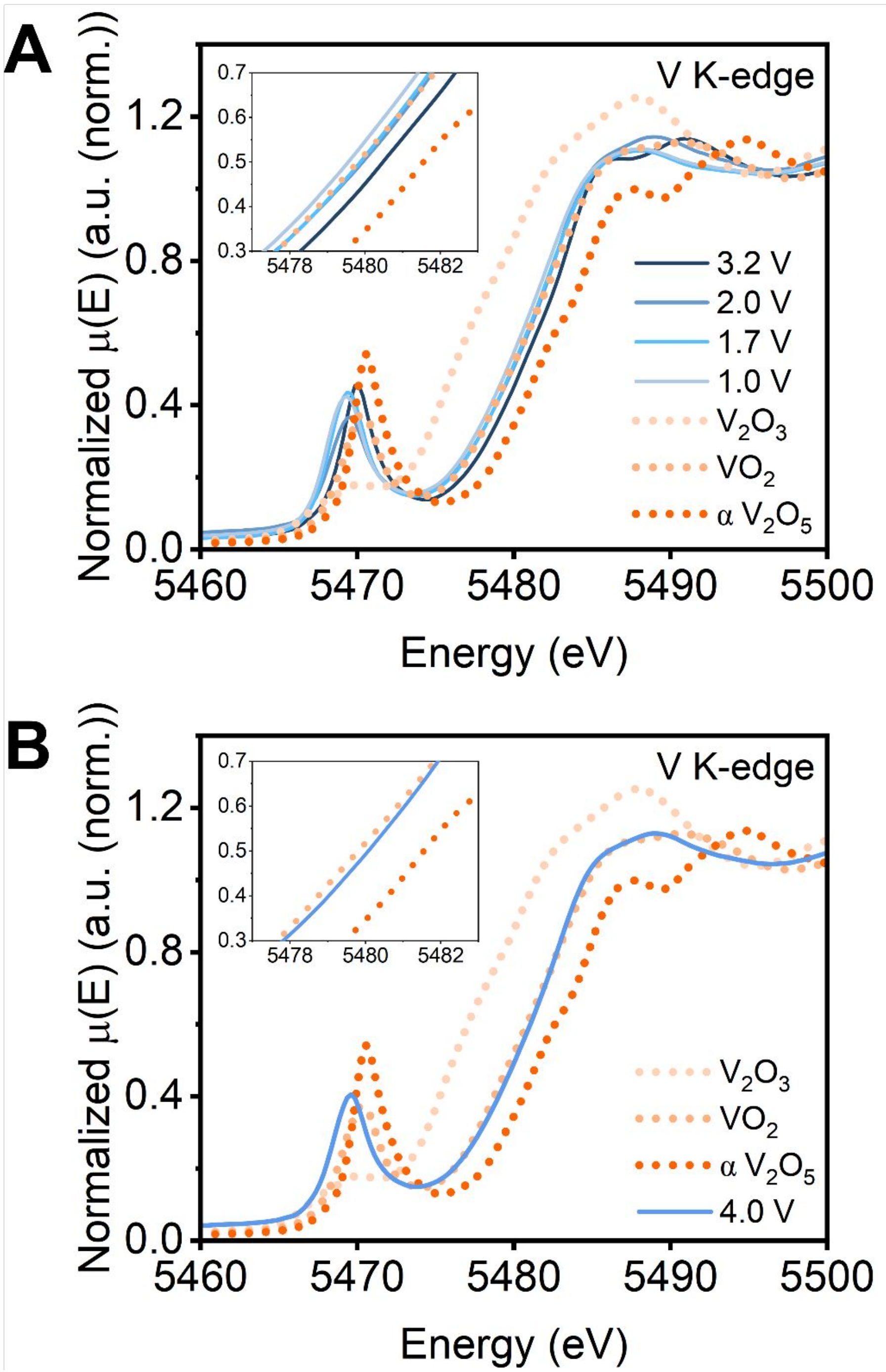

Figure S9. (a) Normalized vanadium K-edge XANES spectra of pristine ζ-V_2O_5 and β-$Na_xV_2O_5$ electrodes at different states of charge (2.0 V, 1.7 V, and 1.0 V) and (b)

β-$Na_xV_2O_5$ electrode at the fully charged state (4.0 V) after the first cycle in the voltage range of 1.0-4.0 V compared to α-V_2O_5, VO_2, and V_2O_3 standards.

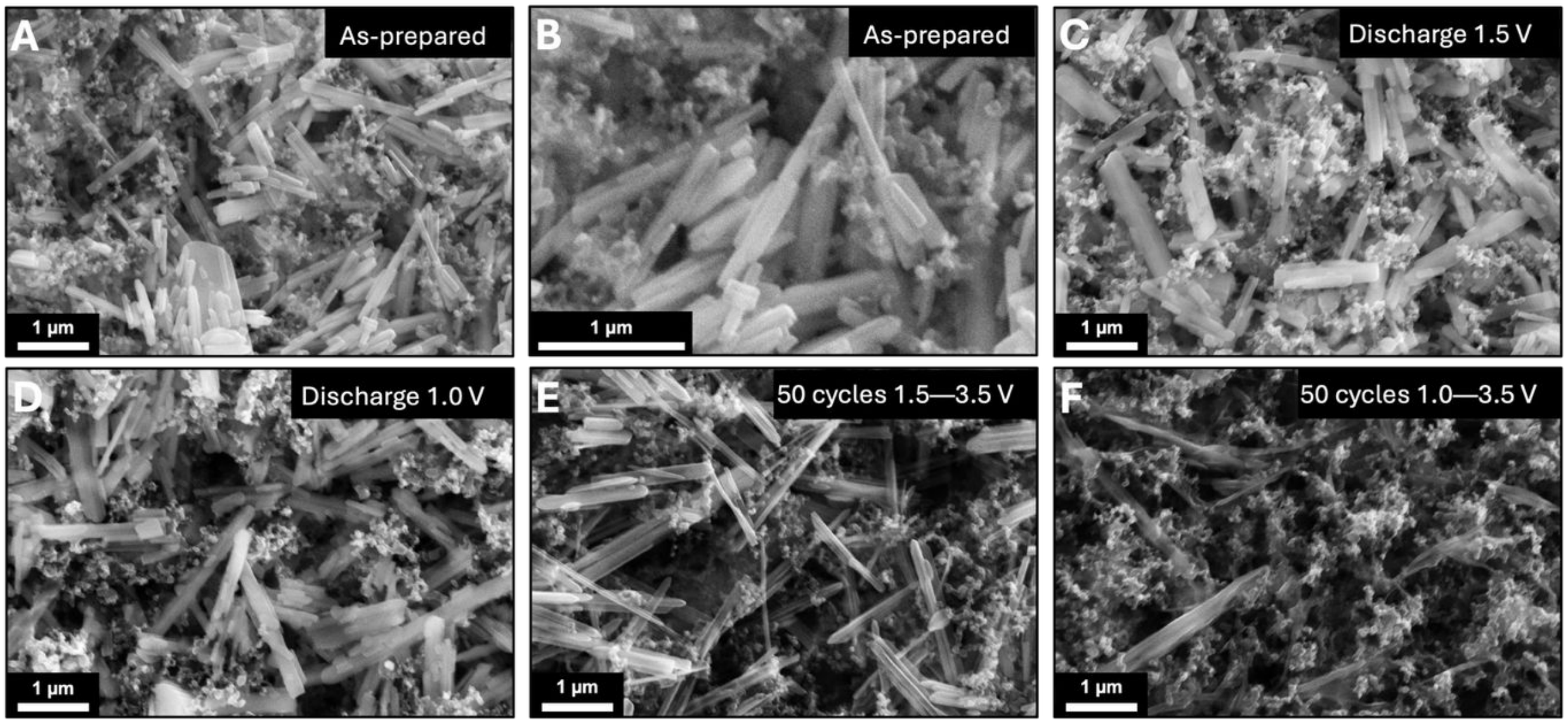

Figure S10. Scanning electron microscopy images of ζ-V_2O_5 electrodes cycled in sodium half-cells acquired at an accelerating voltage of 12 keV. As-prepared ζ-V_2O_5 electrode showing nanowire morphology at a magnification of (a) 15 770x and (b) zoomed-in view in **Figure S10A**. (c) ζ-V_2O_5 electrode after the first discharge process to 1.5 V at a magnification of 15 770x. (d) ζ-V_2O_5 electrode after the first discharge process to 1.0 V at a magnification of 15 770x. (e) ζ-V_2O_5 electrode at the fully charged state (3.5 V) after 50 cycles in a voltage range of 1.5–3.5 V at a magnification of 15 770x. (f) ζ-V_2O_5 electrode at the fully charged state (3.5 V) after 50 cycles in a voltage range of 1.0–3.5 V at a magnification of 15 770x.

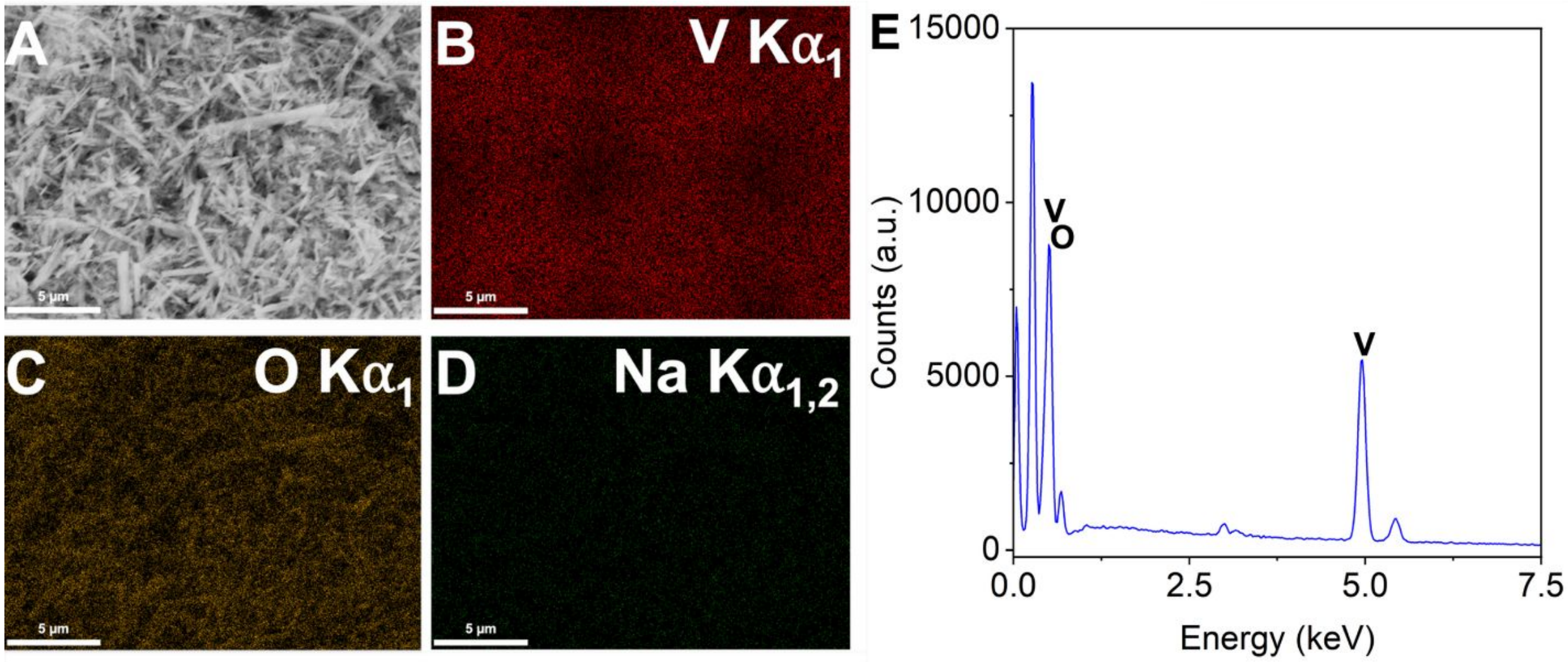

Figure S11. (a) SEM image and corresponding EDX elemental maps of the as-prepared ζ-V_2O_5 electrode acquired at an accelerating voltage of 12 keV at (b) V Kα, (c) O Kα, and (d) Na Kα. (e) EDX map spectrum of the as-prepared ζ-V_2O_5 electrode.

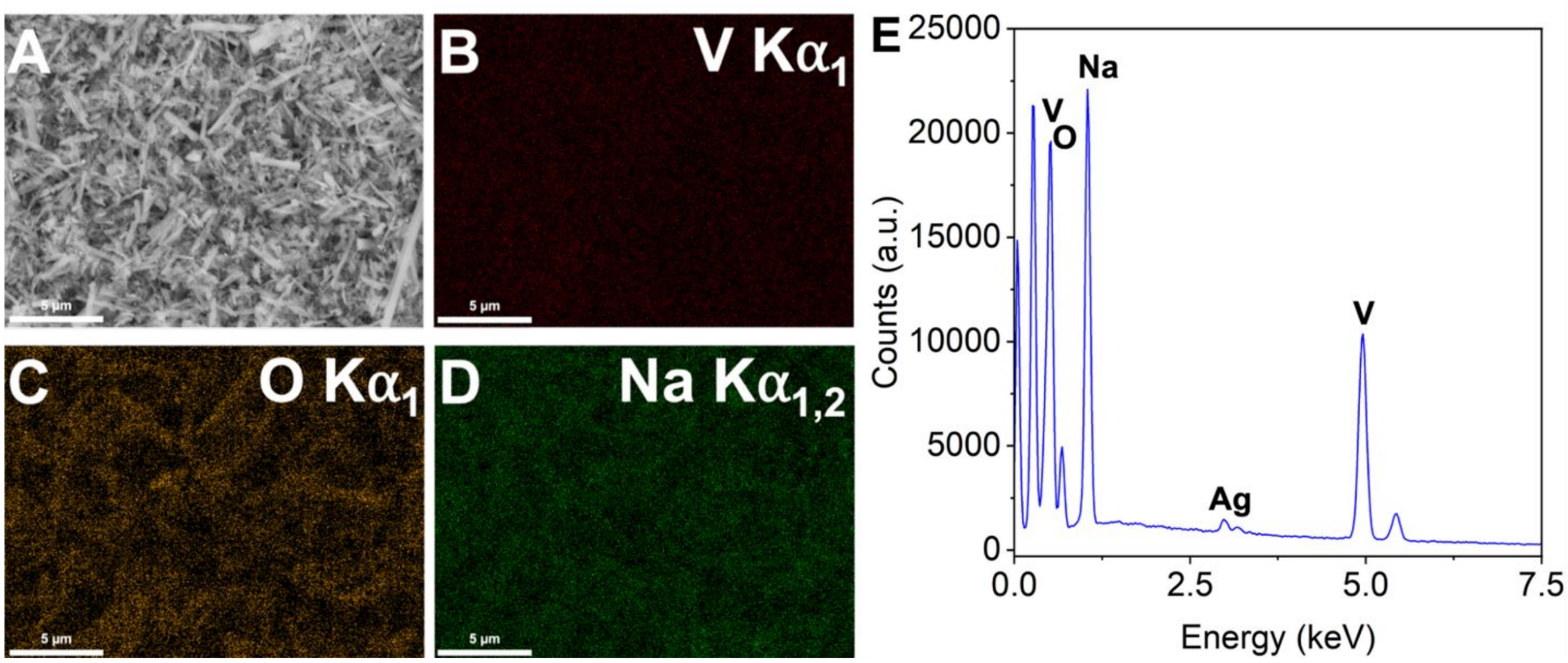

Figure S12. (a) SEM image and corresponding EDX elemental maps of the ζ-V_2O_5 electrode after the first discharge process to 1.5 V, acquired at an accelerating voltage of 12 keV at (b) V Kα, (c) O Kα, and (d) Na Kα. (e) EDX map spectrum of the ζ-V_2O_5 electrode after the first discharge process to 1.5 V.

References:

1. Lee, Y.; Oh, S. M.; Park, B.; Ye, B. U.; Lee, N.-S.; Baik, J. M.; Hwang, S.-J.; Kim, M. H. Unidirectional Growth of Single Crystalline β-$Na_{0.33}V_2O_5$ and α-V_2O_5 Nanowires Driven by Controlling the pH of Aqueous Solution and Their

Electrochemical Performances for Na-Ion Batteries. *CrystEngComm* 2017, 19 (34), 5028-5037, https://doi.org/10.1039/C7CE00781G.
2. Baddour-Hadjean, R.; Bach, S.; Emery, N.; Pereira-Ramos, J. P. The Peculiar Structural Behaviour of β-$Na_{0.33}V_2O_5$ upon Electrochemical Lithium Insertion. *J. Mater. Chem.* 2011, 21 (30), 11296-11305, https://doi.org/10.1039/C1JM11393C.
3. Cai, Y.; Zhou, J.; Fang, G.; Cai, G.; Pan, A.; Liang, S. $Na_{0.282}V_2O_5$: A High-Performance Cathode Material for Rechargeable Lithium Batteries and Sodium Batteries. *J. Power Sources* 2016, 328, 241-249, https://doi.org/10.1016/j.jpowsour.2016.08.016.
4. Orive, J.; Golov, A.; Fernández de Luis, R.; Martínez-Amesti, A.; Flores, M.; Carrasco, J.; Casas-Cabanas, M.; Lisoni, J. Dehydration-Induced δ-to-β Phase Transformation in Vanadium Bronzes: Enabling High-Rate Lithium Storage via Morphology and Redox Modulation. *Chem. Mater.* 2026, https://doi.org/10.1021/acs.chemmater.6c00287.
5. Bach, S.; Baffier, N.; Pereira-Ramos, J.; Messina, R. Electrochemical Sodium Intercalation in $Na_{0.33}V_2O_5$ Bronze Synthesized by a Sol-Gel Process. *Solid State Ionics* 1989, 37, 41-49, https://doi.org/10.1016/0167-2738(89)90006-6.
6. Lu, Y.; Su, N.; Cheng, L.; Liu, J.; Yang, L.; Yang, H.; Yang, Q.; Li, S.; Min, J.; Lei, M. $Na_{0.33}V_2O_5$ Nanosheet@Graphene Composites: Towards High Performance Cathode Materials for Sodium Ion Batteries. *Mater. Lett.* 2016, 183, 346-350, https://doi.org/10.1016/j.matlet.2016.07.128.
7. Kumar, N.; Jerla, N.; Ponis, J.; Banerjee, S.; Sambandamurthy, G. Low-Frequency Noise and Resistive Switching in β-$Na_{0.33}V_2O_5$. arXiv 2025, https://doi.org/10.48550/arXiv.2511.00376.
8. Luo, Y.; Bai, Y.; Mistry, A.; Zhang, Y.; Zhao, D.; Sarkar, S.; Handy, J. V.; Rezaei, S.; Chuang, A. C.; Carrillo, L.; Wiaderek, K.; Pharr, M.; Xie, K.; Mukherjee, P. P.; Xu, B.-X.; Banerjee, S. Effect of Crystallite Geometries on Electrochemical Performance of Porous Intercalation Electrodes by Multiscale *Operando* Investigation. *Nat. Mater.* 2022, 21, 217-227, https://doi.org/10.1038/s41563-021-01151-8.
