# Supplementary material for: Na-Ion Interstitial Site Filling and Structural Evolution in a Tunnel-Structured Vanadium Oxide Insertion Host
Source: ACS Mater Au. 2026 Sep 30;6(6):1659–72. doi: 10.1021/acsmaterialsau.6c00206 (PMC13760625; doi:10.1021/acsmaterialsau.6c00206)

checkCIF/PLATON report

Structure factors have been supplied for datablock(s) zvonai

THIS REPORT IS FOR GUIDANCE ONLY. IF USED AS PART OF A REVIEW PROCEDURE FOR PUBLICATION, IT SHOULD NOT REPLACE THE EXPERTISE OF AN EXPERIENCED CRYSTALLOGRAPHIC REFEREE.

No syntax errors found. CIF dictionary Interpreting this report

Datablock: zvonai

```
Bond precision:   V- O = 0.0011 A          Wavelength=0.71073

Cell:             a=15.3488(5)     b=3.6105(1)        c=10.0803(3)
                  alpha=90         beta=109.874(3)    gamma=90
Temperature:      100 K

                 Calculated                  Reported
Volume           525.35(3)                   525.35(3)
Space group      C 2/m                       C 1 2/m 1
Hall group       -C 2y                       -C 2y
Moiety formula   O15 V6, 0.8(Na)             1.333(Na0.269 O5 V2)
Sum formula      Na0.80 O15 V6               Na0.27 O5 V2
Mr               564.03                      188.09
Dx,g cm-3        3.566                       3.567
Z                2                           6
Mu (mm-1)        5.246                       5.247
F000             533.6                       534.0
F000'            537.54
h,k,lmax         24,5,16                     24,5,16
Nref             1382                        1307
Tmin,Tmax        0.474,0.657                 0.402,1.000
Tmin'            0.253

Correction method= # Reported T Limits: Tmin=0.402 Tmax=1.000
AbsCorr = GAUSSIAN
```

Data completeness= 0.946 Theta(max)= 35.810

R(reflections)= 0.0210(1279) wR2(reflections)= 0.0535(1307)

S = 1.242 Npar= 79

The following ALERTS were generated. Each ALERT has the format
test-name_ALERT_alert-type_alert-level.
Click on the hyperlinks for more details of the test.

Alert level C

```
PLAT041_ALERT_1_C Calc. and Reported SumFormula    Strings  Differ     Please Check
              Calc: Na0.80 O15 V6
              Rep.: Na0.27 O5 V2
PLAT042_ALERT_1_C Calc. and Reported MoietyFormula Strings  Differ     Please Check
              Calc: O15 V6, 0.8(Na)
              Rep.: 1.333(Na0.269 O5 V2)
PLAT077_ALERT_4_C Unit-Cell Contains Non-integer Number of Atoms .     Please Check
```

Alert level G

```
FORMU01_ALERT_1_G  There is a discrepancy between the atom counts in the
            _chemical_formula_sum and _chemical_formula_moiety. This is
            usually due to the moiety formula being in the wrong format.
            Atom count from _chemical_formula_sum:   Na0.27 O5 V2
            Atom count from _chemical_formula_moiety:Na0.358577 O6.665 V2.666
PLAT004_ALERT_5_G Polymeric Structure Found with Maximum Dimension          2 Info
PLAT045_ALERT_1_G Calculated and Reported Z Differ by a Factor ...      0.333 Check
PLAT068_ALERT_1_G Reported F000 Differs from Calcd (or Missing)...     Please Check
PLAT128_ALERT_4_G Alternate Setting for Input Space-group     C2/m       I2/m Note
PLAT302_ALERT_4_G Anion/Solvent/Minor-Residue Disorder (Resd    2)       100% Note
PLAT302_ALERT_4_G Anion/Solvent/Minor-Residue Disorder (Resd    3)       100% Note
PLAT794_ALERT_5_G Tentative Bond Valency for V1        (V)       .       4.95 Info
PLAT804_ALERT_5_G Number of ARU-Code Packing Problem(s) in  PLATON          1 Info
PLAT912_ALERT_4_G Missing # of FCF Reflections Above STh/L=  0.600         75 Note
PLAT969_ALERT_5_G The 'Henn et al.' R-Factor-gap value ..........       3.633 Note
              Predicted wR2: Based on SigI**2  1.47 or SHELX Weight  4.31 Note
```

---

0 **ALERT level A** = Most likely a serious problem - resolve or explain
0 **ALERT level B** = A potentially serious problem, consider carefully
3 **ALERT level C** = Check. Ensure it is not caused by an omission or oversight
11 **ALERT level G** = General information/check it is not something unexpected

5 ALERT type 1 CIF construction/syntax error, inconsistent or missing data
0 ALERT type 2 Indicator that the structure model may be wrong or deficient
0 ALERT type 3 Indicator that the structure quality may be low
5 ALERT type 4 Improvement, methodology, query or suggestion
4 ALERT type 5 Informative message, check

It is advisable to attempt to resolve as many as possible of the alerts in all categories. Often the minor alerts point to easily fixed oversights, errors and omissions in your CIF or refinement strategy, so attention to these fine details can be worthwhile. It is up to the individual to critically assess their own results and, if necessary, seek expert advice.

PLATON version of 23/04/2026; check.def file version of 30/03/2026

Datablock zvonai - ellipsoid plot

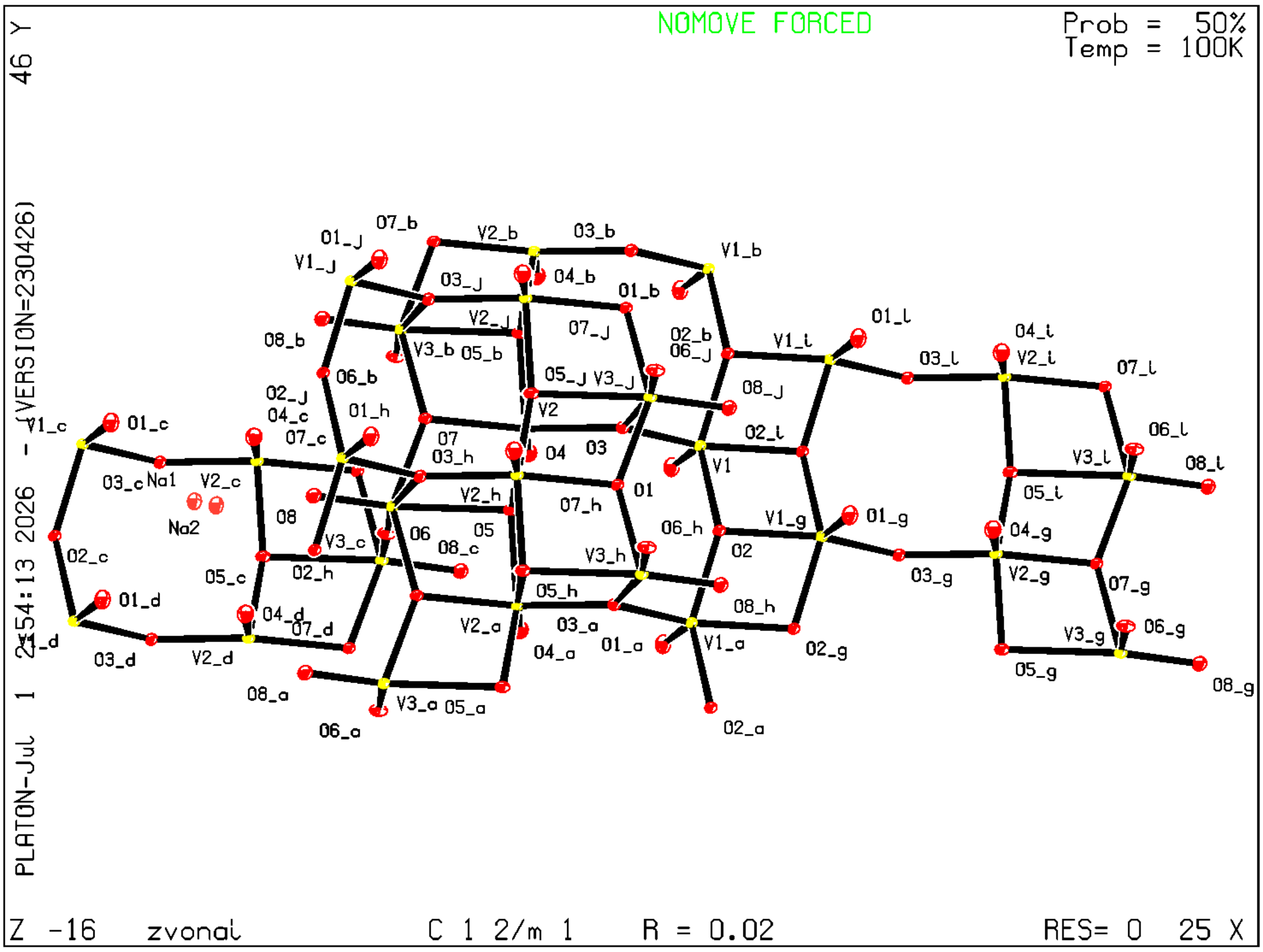

Supplement: Supplementary file 2 [file mg6c00206_si_003.pdf]
